# *In Silico* Identification, Phylogenetic and Bioinformatic Analysis of Argonaute Genes in Plants

**DOI:** 10.1155/2014/967461

**Published:** 2014-09-15

**Authors:** Khaled Mirzaei, Bahman Bahramnejad, Mohammad Hasan Shamsifard, Wahid Zamani

**Affiliations:** ^1^Department of Agricultural Biotechnology, Faculty of Agriculture, University of Kurdistan, Sanandaj 66177-15175, Iran; ^2^Laboratory of Alpine Ecology, Joseph Fourier University, 38041 Grenoble, France

## Abstract

Argonaute protein family is the key players in pathways of gene silencing and small regulatory RNAs in different organisms. Argonaute proteins can bind small noncoding RNAs and control protein synthesis, affect messenger RNA stability, and even participate in the production of new forms of small RNAs. The aim of this study was to characterize and perform bioinformatic analysis of Argonaute proteins in 32 plant species that their genome was sequenced. A total of 437 Argonaute genes were identified and were analyzed based on lengths, gene structure, and protein structure. Results showed that Argonaute proteins were highly conserved across plant kingdom. Phylogenic analysis divided plant Argonautes into three classes. Argonaute proteins have three conserved domains PAZ, MID and PIWI. In addition to three conserved domains namely, PAZ, MID, and PIWI, we identified few more domains in AGO of some plant species. Expression profile analysis of Argonaute proteins showed that expression of these genes varies in most of tissues, which means that these proteins are involved in regulation of most pathways of the plant system. Numbers of alternative transcripts of Argonaute genes were highly variable among the plants. A thorough analysis of large number of putative Argonaute genes revealed several interesting aspects associated with this protein and brought novel information with promising usefulness for both basic and biotechnological applications.

## 1. Introduction

Pathways of gene silencing and small regulatory RNAs such as miRNAs (microRNAs) and siRNAs (short interfering RNAs) are widespread in almost all eukaryotic organisms [1, 2]. These pathways are known to act in development, heterochromatin formation, regulation of gene expression at transcription, posttranscription, and translation level, or mRNA stability [3–5]. Biochemical RNA silencing and small regulatory RNAs processes are mediated by a number of proteins which include Dicers, Argonautes, and RNA-dependent RNA polymerases [3, 6]. Investigations in eukaryotes have revealed that these proteins are encoded in a family with variable number of genes [1, 6–9].

In the pathways of gene silencing and small regulatory RNAs, Argonaute proteins have key catalytic role in translational repression or cleavage. These proteins are ~100-kD, highly basic proteins and share the domain structure that comprises an N terminal, PAZ, Mid, and a C-terminal PIWI domain [6, 10, 11]. The PAZ domain (~100 aa) facilitates binding of 3′ end of siRNA, while the PIWI domain binds the 5′ end of siRNA. This domain has marked similarity with RNaseH family of ribonucleases which is carried out by an active site usually carrying an Asp-Asp-His (DDH) motif and it possesses the catalytic amino acid residues required for endonucleolytic cleavage of the target RNA but in some of the Argonaute proteins (HsAgo3) which have DDH domain but do not appear to have slicer activity, it suggests that the presence of a DDH motif does not necessarily imply slicer activity [11–14].

At least three subfamilies of Argonaute proteins have been identified in eukaryotes: the Argonaute subfamily present in plants, animals, and yeasts, the PIWI subfamily found only in animals, and the worm-specific Argonaute or WAGO subfamily present in* C*.* elegans*. Members of both Argonaute and PIWI subfamilies possess the characteristic DDH metal binding signature residues in their PIWI domains, while most of the WAGO proteins lack them [7, 15, 16]. The PIWI proteins are expressed specifically in the germline cells and are known to interact with a subset of small RNA called PIWI-interacting RNA that are longer (26–31 nt) than siRNA and miRNA (21–24 nt). PIWI class performs the small RNA in animal germ cells but in plants it is performed by member(s) of the Argonaute class [13, 17].

The Argonaute protein family was first identified in plants, and members are defined by the presence of PAZ (PIWI-Argonaute-Zwille) and PIWI domains. Argonaute proteins are highly conserved between species and many organisms encode multiple members of these genes. Plant Argonaute proteins are evolutionarily conserved and in the phylogenic analysis group divided into three clades [13].

The numbers of Argonaute genes vary in different species, ranging from 1 in the fission yeast* Schizosaccharomyces pombe* to 27 in the nematode worm* C*.* elegans* [7, 11, 13, 17]. There are eight Argonaute genes in mammals and five genes in the* D*.* melanogaster* genome [7, 13]. Argonaute proteins are ubiquitously expressed and bind to siRNAs or miRNAs to guide posttranscriptional gene silencing either by destabilization of the mRNA or by translational repression. Although various aspects of Argonaute function have been identified, many Argonaute proteins are still poorly characterized [11, 13, 15–17]. At present, some reports reveal genome-wide organization and expression analysis of plant Argonaute gene family in* Oryza sativa*,* Zea mays*,* Arabidopsis thaliana*,* Medicago truncatula,* and* Solanum lycopersicum* [12, 18–20]. Numbers of Argonaute genes in plant such as* A*.* thaliana* are 10 members with some of them being characterized with respect to biological function. Argonaute1 and Argonaute10 are involved in shoot meristem, Argonaute4 is involved in RNA-directed DNA methylation and silencing of a small class of transposons, and Argonaute7 is involved in the juvenile-adult transition in vegetative development [13, 21]. Plant reproduction also requires RNAi machinery, in which Argonaute1 acts in effecting the full expression of LEAFY (*LFY*), APETALA1 (*AP1*), and AGAMOUS (*AG*), encoding transcription factors to determine meristem identity, flowering transition, and/or flower organ identity. In addition, Argonaute1 plays a central role in the posttranscriptional gene silencing of CURLYLEAF (*CLF*), encoding a Polycomb group protein that maintains the repression of both KNOTTED-like homeobox (*KNOX*) genes and homeotic genes AG and APETALA3 (*AP3*) in vegetative leaves, and in pollen development [21, 22]. Argonaute10 is initially expressed throughout the embryo but becomes limited to the provascular strands and the adaxial sides of the cotyledons at about the globular stage.

The completion of whole genome sequencing (WGS) of important crops has opened a new dimension of genetic data mining, which will ultimately impact agricultural and industrial use of these crops in upcoming years. Sequences derived from large-scale sequencing projects are informative in functional genomics research and provide the opportunity to genome-wide scan of gene families and comprehensive comparative genome study is essential for understanding the evolution and function of each gene family in plants. Although studies on Argonaute have been covered in different biological systems, the availability of genome sequences of more organisms has provided significant information about newly sequenced genes encoding Argonaute proteins in higher plants. This represents an avenue for gene discovery and functional comparative genomics studies. In this study, we report on the phylogenetic relationship and the structural and functional characterization of Argonaute gene subfamilies in higher plants. The aim of this investigation is characterization and bioinformatic analysis of Argonaute protein in 32 plant species and* A*.* thaliana* as a reference.

## 2. Material and Methods

Argonaute genes of 32 plants were verified by Blastp searches (according to default program settings) using Arabidopsis thaliana* AtAGO1* to* AtAGO10*. Likewise, an *E*-value threshold (the number of times that a match, or a better match, occurs by chance within the database of 0 to 1*e* − 50) was used.

Evaluation of Argonaute candidates was done based on the identification of domains in the NCBI Conserved Domains Database (CDD) that is specific for the different proteins: PAZ (Cd02846) of superfamily (Cl00301), MID (5′ RNA guide strand anchoring site), PIWI (Cd04657) of superfamily (Cl00628), and total protein (PLN03202). The domains were identified as part of the NCBI web-based Blast interface which includes an RPS-Blast search versus the position-specific scoring matrices in CDD (v3. 10-44354 PSSMs) [23]. The obtained sequences were also subjected to reciprocal Blastp searches, ensuring that they indeed were most similar to proteins of the respective family. Most searches were conducted using the nonredundant protein database at NCBI and phytozome of June 2013 (http://www.phytozome.net/).

Protein alignments were performed using CLUSTALW [24], with manual adjustment/editing using BioEdit [25]. Argonaute genes were prefixed with the corresponding genus and species initials. For phylogenetic analysis of conserved domains, sequences were trimmed so that only the relevant protein domains remained in the alignment. Phylogenetic trees were constructed using MEGA 4 software [26] based on the sequence of Argonaute to determine the distribution and evolutionary trend of Argonaute in plants using the neighbor-joining (NJ) method with 100 bootstrapping replicates.

Three-dimensional structure of proteins was performed by the PHYRE^2^ server [27] and three-dimensional structures were received as the PDB format. Then this format was fed to YASARA [28] software to draw three-dimensional structure, c-terminal, n-terminal, and also three domains PAZ, MID, and PIWI.

### 2.1. Expression Profiles Investigation of Plant Argonaute Genes

Transcript levels of* Arabidopsis thaliana, Oryza sativa, Medicago truncatula, Vitis vinifera, Glycine max, Populus trichocarpa, Prunus persica, Malus domestica,* and Aquilegia coerulea Argonaute genes were analyzed by multiple methods. First, EST mining was performed in the NCBI EST database (http://www.ncbi.nlm.nih.gov/dbEST/) using megablast tool. Parameters of searching were as follows: maximum identity > 95%, length > 200 bp, and *E* value < 10^−10^. Secondly, expression data in the PlantGDB and MAGI databases, including EST, cDNA, and PUTs (PlantGDB unique transcripts), were retrieved by the GDB genome browser tool. Third, EST mining was performed in the DFC-Plant Gene Indices EST database (http://compbio.dfci.harvard.edu/tgi) using identifiers or keywords and expression summary tools.

### 2.2. Mapping Argonaute on Multiplant Chromosomes

Chromosomal position of Argonaute genes of several plants including* Arabidopsis thaliana, Brachypodium distachyon*,* Glycine max*,* Medicago truncatula*,* Populus trichocarpa*, and* Vitis vinifera* was plotted using the NCBI map viewer tool (http://www.ncbi.nlm.nih.gov/mapview/) and for* Cucumis sativus* was plotted using cucumber genome database (http://cucumber.genomics.org.cn/page/cucumber/index.jsp) map viewer tool.

## 3. Results

### 3.1. Protein Sequence Collection and Classification for Argonaute Gene Families

The first step of our analysis was to identify all Argonaute genes from 32 plant species that their genome was sequenced (Table 1). To identify Argonaute genes and their putative encoded polypeptides present in* Arabidopsis* genome, initially, keyword search of Argonaute against A. thaliana genome database was performed (http://www.ncbi.nlm.nih.gov/). It was found that 10 members had been annotated as Argonaute genes displayed in numbers 1–10 (ref* A. thaliana Argonaute*).

*A. thaliana Argonaute* genes were used in phytozome database [29] as query using the Blastp search engine. In most cases, whenever significant similarity to Argonaute sequence was identified in other species, the genomic sequence was excised and homology-based gene predictions were performed using the most similar query as a guide. Blastp analysis was carried out to search against a database from thirty two species. For most of the gene families, an *E* value cut off of *e* − 30 was used. The results of our extensive database searches are summarized in Table 1. The numbers of identified putative Argonaute genes varied from 6 in* Carica papaya* to 24 in* Panicum virgatum*. Some of the Argonaute genes loci had alternative transcripts. In this study, only the transcript with most conserved domains, which is the transcript with lowest *E*-value of domain examination, was selected. Finally, 437 Argonaute genes were obtained for all 32 plants. These Argonaute genes were designated by AGO.

### 3.2. Phylogeny

To examine the relationships of Argonaute proteins and investigate the evolutionary history of this protein family among the plants, phylogenetic trees were constructed using MAGE v4.0 program by the N-J method. Because of large numbers of studied plant species and large numbers of putative Argonaute proteins, phylogenetic tree for Argonaute proteins was drawn in the separate section. In order to visualize phylogenetic relationships clearly, shortened gene names were used on the phylogenetic trees. We divided plants into four groups and division was based on a phylogeny tree of species in phytozome v9.1 website.

**Figure 1:**
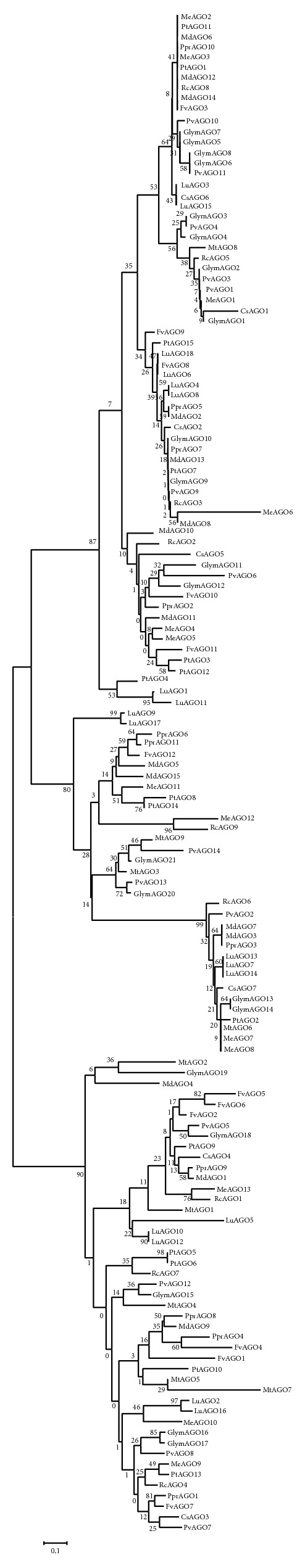
Phylogenetic tree of the Argonaute protein related to the Fabidae groups which consist of* Manihot esculenta, Ricinus communis, Linum usitatissimum, Populus trichocarpa, Medicago truncatula, Phaseolus vulgaris, Glycine max, Cucumis sativus, Prunus persica, Malus domestica,* and* Fragaria vesca* sequences.

The first phylogenetic analysis of 144 Argonaute proteins was done for Fabidae group. Numbers of Argonaute protein genes in each species are shown in Table 1. Phylogenic analysis divided Argonaute genes of these plants into three classes 1, 2, and 3. Class 1 which contains 66 sequences was classified into two subclasses. Class 2 includes 33 sequences which also subdivided into three subclasses. Third class has 45 sequences and subdivided into two subclasses (Figure 1).

**Figure 2:**
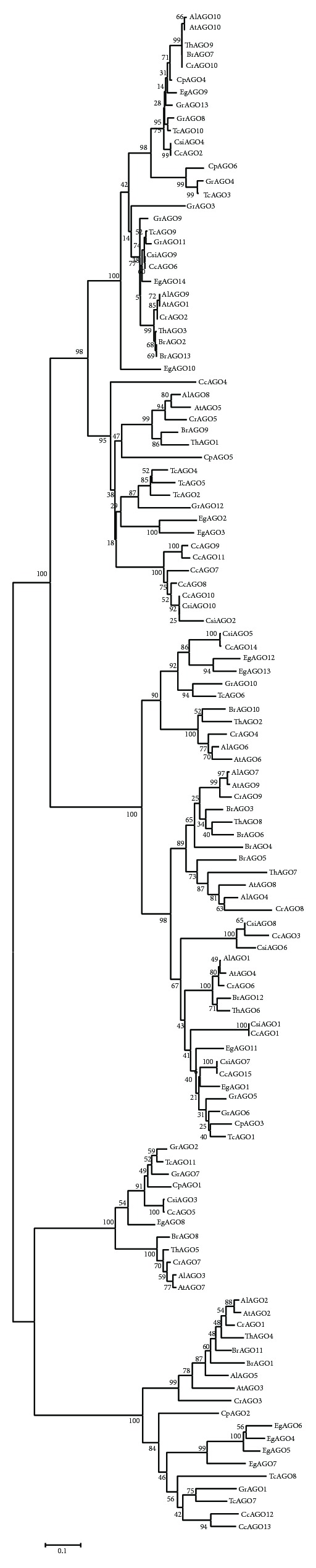
Phylogenetic tree of the Argonaute protein related to the Malvidae group which consists of* A. thaliana, A. lyrata, Capsella rubella, Brassica rapa, Thellungiella halophila, Carica papaya, Gossypium raimondii, Theobroma cacao, Citrus sinensis, Citrus clementina,* and* Eucalyptus grandis AGO* sequences.

The second group consists of 121 sequences and belongs to Malvidae group. Phylogenic analysis divided Argonaute genes of this group into three classes. Class 1 which contains 49 sequences was classified into two subclasses. Class 2 includes 41 sequences which also divided into three subclasses. Third class has 31 sequences and was classified into two subclasses like first phylogeny (Figure 2).

**Figure 3:**
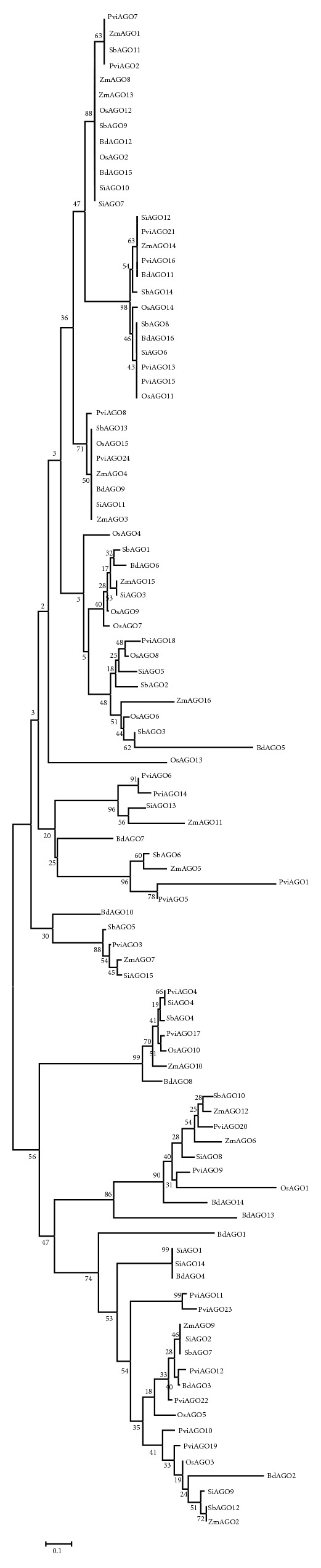
Phylogenetic tree of the Argonaute protein related to the Grass group which consists of* Sorghum bicolor, Zea may, Setaria italica, Panicum virgatum, Oryza sativa,* and* Brachypodium distachyon AGO* sequences.

Third phylogenetic analysis related to 100-sequence Grass group. Phylogenic analysis divided Argonaute genes of this group into three classes. Class 1 which contains 60 sequences was classified into two subclasses. Class 2 includes 13 sequences which also divided into two subclasses. Third class has 20 sequences and was classified into two subclasses like phylogenies one and two (Figure 3).

**Figure 4:**
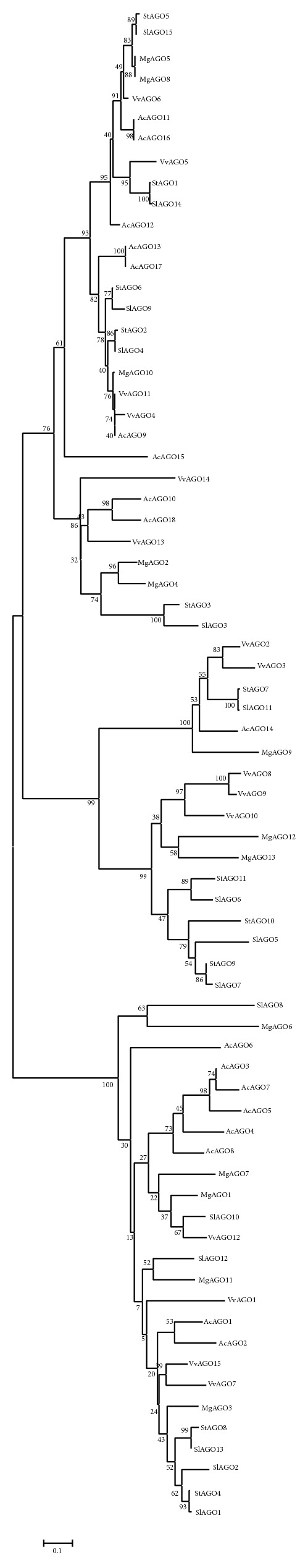
Phylogenetic tree of the Argonaute protein related to* Vitis vinifera, Solanum tuberosum, Solanum lycopersicum, Mimulus guttatus,* and* Aquilegia coerulea* plant separately.

Fourth phylogenetic analysis of the other plants consists of 72 sequences. Phylogenic analysis divided Argonaute genes of these plants into three classes. Class 1 which contains 30 sequences was classified into two subclasses. Class 2 includes 17 sequences which also divided into three subclasses. Third class has 25 sequences and was classified into two subclasses like first phylogeny (Figure 4).

### 3.3. Analyses of Conserved Region and Sequence of Argonaute Proteins

**Figure 5:**
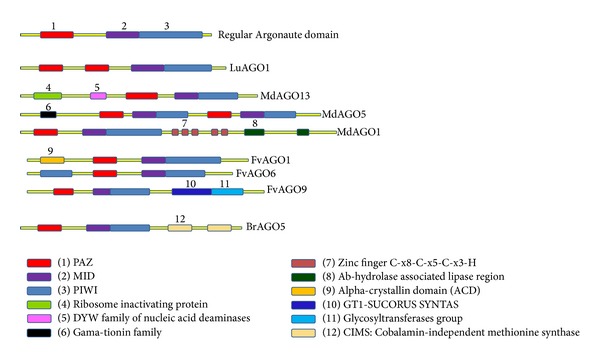
Domain architecture variations in plant Argonautes. The protein domains were obtained using the Conserved Domains Database (CDD) database of NCBI. The Argonaute protein regular domains domains PAZ (red), MID (violet) and PIWI (blue) are shown in addition to other unregular domains.

Bioinformatics analysis of Argonaute protein plant was done using the Conserved Domains Database (NCBI) and domains sequences were drawn for each group and placed side by side. Argonaute proteins usually have PAZ, MID, and PIWI domains and all of participated sequences in our investigation had PAZ, MID, and PIWI domains but length and location of these domains in each sequence were variable (Figure 5).

Structural analysis of the Argonaute protein sequence in studied plants revealed that all of the sequences that had similar structure and location of domains in the protein are identical; therefore, it seems that all of these proteins have been highly conserved and operate the same activities. Results showed that Argonaute protein contained *α*-helix and *β*-folding, belonging to a hybrid protein structure and creating the suitable location for performing the activity to synthesize the specific binding pocket that anchors the characteristic two-nucleotide 3′ overhang that results from digestion of RNAs by RNase III (a step in the processing of small RNAs) or this structure has proper location for implicated MID domain in protein-protein interactions (Figure 7).

### 3.4. Unusual Domains

Our analysis showed that most of the plant AGO examined encode PAZ, MID, and PIWI domains. However, we noticed anomalies in the domain organization as well. LuAGO1 has two PAZ domains and one MID and PIWI domain. Besides three conserved domains, MdAGO13 encodes two more domains ribosome-inactivating protein and DYW family of nucleic acid deaminases which are located before conserved domains. MdAGO5 had two complete groups of PAZ, MID, and PIWI domains that are placed after gamma-thionin family domain (Figure 5). MdAGO1 as well as regular domains had two extra domains which are placed after these domains and their names are Zinc finger C-x8-C-x5-C-x3-H type and ab-hydrolase associated lipase region, respectively (Figure 5). Also FvAGO1 in addition to PAZ, MID, and PIWI domains had alpha-crystallin domain (ACD) of alpha-crystallin-type small (s) heat shock proteins (Hsps) that is located in front of PAZ domain. Except for regular domains, FvAGO9 has GT1-SUCORUS SYNTAS domain additionally which is located after PIWI was placed and Glycosyltransferases catalyze the transfer of sugar moieties from activated donor molecules to specific acceptor molecules, forming glycosidic bonds. FvAGO6 in addition to PAZ and MID had two PIWI domains. Sequence of BrAGO5 had regular domains as well as two CIMS N terminals like domains which are located after the PIWI domain (Figure 5).

### 3.5. Alternative Transcripts

Numbers of alternative transcripts of Argonaute gene in the plant were highly variable. Argonaute genes in* Ricinus communis, Linum usitatissimum, Populus trichocarpa, Malus domestica, Fragaria vesca, Arabidopsis lyrata, Capsella rubella, Vitis vinifera, Brassica rapa, Carica papaya, Mimulus guttatus,* and* Solanum lycopersicum* did not have alternative transcripts, but* Manihot esculenta, Medicago truncatula, Phaseolus vulgaris, Glycine max, Cucumis sativus, Prunus persica, Arabidopsis thaliana, Thellungiella halophila, Gossypium raimondii, Theobroma cacao, Citrus sinensis, Citrus clementina, Eucalyptus grandis, Solanum tuberosum, Aquilegia coerulea, Sorghum bicolor, Zea mays, Setaria italica, Panicum virgatum, Oryza sativa,* and* Brachypodium distachyon* Argonaute genes had different alternative transcripts (Table 1). Argonaute genes loci in* Aquilegia coerulea* had highest alternative transcripts number compared to that of other studied plants. AGO15 gene locus in the* Aquilegia coerulea* with 17 different transcripts had the highest Argonaute alternative transcripts (Table 1).

### 3.6. Chromosome Location

**Figure 6:**
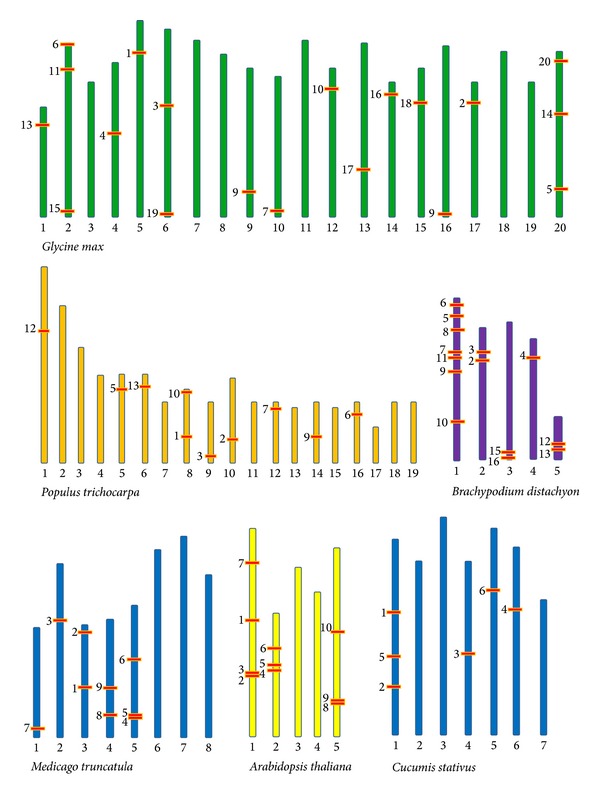
Chromosome distribution and expansion pattern of the Argonaute gene in the* A. thaliana, Brachypodium distachyon, Cucumis sativus, Glycine max, Medicago truncatula, and Populus trichocarpa*.

**Figure 7:**
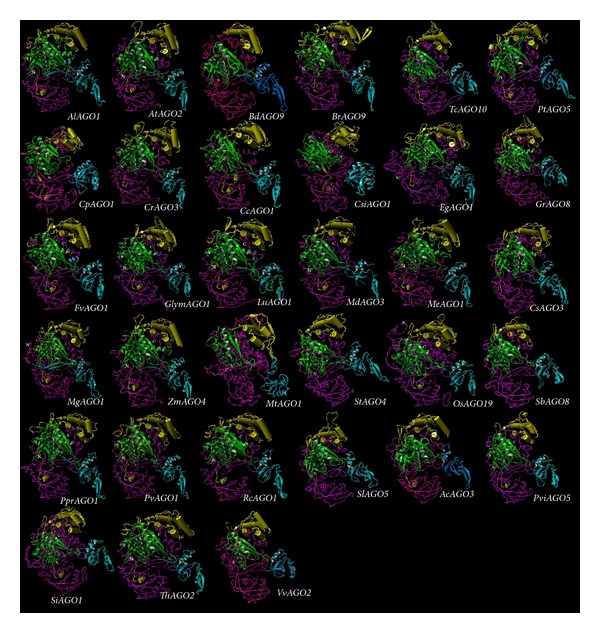
Predicted Argonautes protein fold of* AlAGO1*,* AtAGO2*,* BdAGO9*,* BrAGO4*,* TcAGO10*,* PtAGO5*,* CpAGO1*,* CrAGO3*,* CcAGO1*,* CsiAGO1*,* EgAGO1*,* GrAGO8*,* FvAGO1*,* GlymAGO1*,* LuAGO1*,* MdAGO3*,* MeAGO1*,* CsAGO3*,* MgAGO1*,* ZmAGO4*,* MtAGO1*,* StAGO4*,* OsAGO19*,* PprAGO1*,* PvAGO1*,* RcAGO1*,* SlAGO5*,* SbAGO8*,* AcAGO3*,* SiAGO1*,* ThAGO2*,* VvAGO2,* and* PviAGO5*.

In order to determine the synteny between Argonaute genes in the studied plants the physical locations of Argonaute genes were depicted using NCBI (Figure 6). Physical locations of small number of Argonaute genes such as* GlymAGO8, GlymAGO12, GlymAGO21, PtAGO4, PtAGO8, PtAGO11, PtAGO14, PtAGO15, BdAGO1, BdAGO14,* and* CsAGO7* were not found in the NCBI database. In* A. thaliana AGO1*,* AGO2*,* AGO3*, and* AGO7* were located on chromosome 1,* AGO4*,* AGO5*, and* AGO6* were on chromosome 2, and* AGO8*,* AGO9*, and* AGO10* were on chromosome 5. However, no Argonaute gene was located on chromosomes 3 and 4. In* Brachypodium distachyon AGO6*,* AGO10*,* AGO9*,* AGO3*,* AGO5,* and* AGO7* were on chromosome 1,* AGO13* and* AGO11* were on chromosome 2,* AGO2* and* AGO4* were on chromosome 3,* AGO12* was on chromosome 4, and* AGO1* and* AGO14* were on chromosome number 5. In the* Cucumis sativus AGO1*,* AGO2,* and* AGO3* were located on chromosome 1,* AGO5* was on chromosome 4,* AGO4* was on chromosome 5, and* AGO6* was on chromosome 6. However no Argonaute gene was located on chromosomes 2, 3, and 7 (Figure 6). In the* Glycine max AGO12* was on chromosome 1,* AGO5*,* AGO11,* and* AGO14* were on chromosome 2,* AGO7* was on chromosome 4,* AGO6* was on chromosome 5,* AGO8* and AGO18 were on chromosome 6,* AGO2* was on chromosome 9,* AGO4* was on chromosome 10,* AGO10* was on chromosome 12,* AGO16* was on chromosome 13,* AGO17* was on chromosome 15,* AGO15* was on chromosome 14,* AGO1* was on chromosome 16,* AGO9* was on chromosome 17, and* AGO3*,* AGO13,* and* AGO19* were on chromosome 20. However no Argonaute gene was located on chromosomes 3, 7, 8, 11, 18, and 19. In the* Medicago truncatula AGO4* was on chromosome 2,* AGO8* was on chromosome 3,* AGO3* was on chromosome 4,* AGO5*,* AGO6,* and* AGO7* were on chromosome 5, and* AGO1* was on chromosome 8 (Figure 6). In the* Populus trichocarpa* which has 19 chromosomes and according to the chromosome gene map location of Argonaute gene on* Populus trichocarpa* chromosome* AGO5* was on chromosome 1,* AGO10* was on chromosome 6,* AGO11* and* AGO2* were on chromosome 8,* AGO6* was on chromosome 9,* AGO3* and* AGO8* were on chromosome 10,* AGO1* was on chromosome 12,* AGO12* was on chromosome 14, and* AGO9* was on chromosome 16. However no Argonaute gene was located on chromosomes 2, 3, 4, 5, 7, 11, 13, 15, 17, 18, and 19. In the* Vitis vinifera AGO12* was on chromosome 1,* AGO2* was on chromosome 5,* AGO3* and* AGO14* were on chromosome 6,* AGO1* and* AGO8* were on chromosome 8,* AGO9*,* AGO10,* and* AGO11* were on chromosome 10,* AGO4* was on chromosome 11,* AGO7* was on chromosome 12,* AGO6* was on chromosome 13, and* AGO5* was on chromosome 17. However no Argonaute gene was located on chromosomes 2, 4, 7, 9, 14, 15, 16, 18, and 19. In the* Vitis vinifera AGO13* was on chromosome 3 but location of this gene is not distinct on chromosome 3 (Figure 6).

### 3.7. Expression Profiles

Expressed sequence tags (EST) data can provide valuable information about gene expression research. Expression profiles of Argonaute genes were investigated by multiple strategies in this study (Table 2). EST mining results indicated that major Argonaute genes were expressed in checked tissues and organs. However, expression evidences of some Argonaute genes were detected in only one tissue or organ. Examination of the expression profiles of Argonaute proteins in some of the plants indicated that these proteins have high expression in the seed, leaf, root, and shoot in the studied plants. In the* A. thaliana* the expression of seven Argonaute genes in different tissues including leaf, root, flower, seed, hypocotyls, and ovule was studied. Most of the* A. thaliana* Argonaute genes were expressed in the seed tissue. The lowest numbers of* A. thaliana* Argonaute genes were expressed in hypocotyls tissue. Five Argonaute genes were studied in the rice and their expression was detected in the root, leaf, shoot, flower, seed, pollen, callus, panicle, and ovule. All rice Argonaute genes were expressed in the callus and only one gene was expressed in pollen. In the* Medicago truncatula* expression profiles of six Argonaute genes were studied in the root, leaf, flower, seed, stem, cotyledon, and callus. Most of genes were expressed in the root and only one Argonaute was expressed in callus. In the* Vitis vinifera* only one Argonaute gene was expressed in the seed and pericarp. Data for six Argonaute genes in* Glycine max* in different organs including leaf, shoot, flower, seed, hypocotyl, and cotyledons were studied. All of Argonaute genes transcripts were detected in the seed and only one gene was expressed in hypocotyls. Information related to* Populous trichocarpa* Argonaute expression was available for five Argonautes in different organs including root, leaf, stem, cambium, and buds. All of genes were expressed in leaf and stem and three Argonaute genes were expressed in cambium and bud. In* Prunus persica* only a few pieces of information related to expression of Argonaute protein existed which this Argonaute protein expressed in the fruit and mesocarp. Information about expression of Argonaute genes in* Malus domestica* only existed for three genes that were expressed in the leaf, flower, buds, and fruit. All genes were detected in fruit. The EST data were available for 12 Argonaute sequences for* Aquilegia coerulea*. Number of genes expressed in root, leaf, shoot, and flower was approximately equal.

In general, expression was determined for the 28 Argonaute genes in root, 30 in the leaf, 18 in the shoot, 24 in the flower, 20 in the seed, 8 in the stem, 8 in the callus, 7 in the cotyledon, 6 in the bud, 4 in the ovule, 4 in the fruit, 3 in panicle, 3 in the cambium, 2 in the hypocotyl, 2 in the pistil, 1 in the pollen, 1 in the pericarp, and 1 in mesocarp. In general most expression was related to leaf and seed; lowest number of genes was expressed in the pollen, pericarp, and mesocarp (Table 2). Data showed that in each organ or tissue at least one Argonaute was expressed.

### 3.8. Biochemical Characters

The average Argonaute sequence length was 972 amino acids, the longest length was related to MdAGO1 with 2583 aa, and the shortest length was related to PvAGO1 with 376 aa. The average of molecular mass was 108 kD, the highest was for MdAGO1 with 288 kD, and the lowest was for PvAGO1 with 42 kD. Average isoelectric point of the proteins was 9.39, the lowest was related to MgAGO7 with 6.38, and the highest was for PviAGO18 with 9.96. Average of aliphatic index was 80, the highest was related to GmAGO4 with 92.57, and the lowest was for PviAGO1 with 66.986. Average counts of hydrophobic and hydrophilic residue were 0.474 and 0.270, respectively. The highest count of hydrophobic was for MtAGO6 with 0.432 and the highest count of hydrophilic was for SbAGO5 with 0.527. The lowest count of hydrophobic was for VvAGO10 with 0.234 and the lowest count of hydrophilic was for MtAGO6 with 0.31. Average count of charged residues was 0.099 and 0.128 for negative and positive, respectively. Average alpha helix was 27, the highest was related to MdAGO1 with 71 alpha helices and the lowest was related to BdAGO1 with 11 alpha helices. Average beta strand was 38, the highest was related to MdAGO1 with 99 beta strands and the lowest related to VvAGO3 and VvAGO4 with 18 beta strands (Table 3 Supplementary data available online at http://dx.doi.org/10.1155/2014/967461).

## 4. Discussion 

Classification and phylogenetic analysis of Argonaute proteins among the 32 plants showed that these proteins have high level of conservation. All of the phylogenetic trees were classified in the same manner and consisted of three subclasses. Similar results were obtained for each of plant Argonaute proteins that were classified into three classes. Most of the sequences had PAZ, MID, and PIWI domains and only variation among these sequences was related to length and location of domains in each sequence. Structural analysis of the sequences of Argonaute protein revealed that all of the sequences had similar structure and location of domains in the protein. This demonstrates that all genes are highly conserved during evolution and perform similar functions.

Plant Argonaute showed a wider range of biochemical characters such as molecular weight and length compared to previous studies [11, 15, 30]. Among plants Argonaute proteins the higher average of lengths and weight belonged to Brassica rapa and was 1024 aa 139 kDa, respectively. The isoelectric point is the pH at which a particular molecule or surface carries no net electrical charge. Count of hydrophobic and hydrophilic residue and count of charged residues showed small variation. The aliphatic index of a protein is a measure of the relative volume occupied by aliphatic side chain and an increase in the aliphatic index increases the thermostability of globular Protein. Different amount of this factor may related to different behavior of Argonautes in terms of thermostability. Numbers of beta strands and alpha helices in these proteins were different which may be related to size of each sequence and amino acids content and secondary structure of proteins.

One of the important results of this investigation was finding of unusual domains in some of Argonaute proteins. All of the regular Argonaute proteins had only one PAZ, MID, and PIWI domain, but LuAGO1 had two PAZ domains, one MID, and one PIWI domain. FvAGO7 in addition to PAZ and MID had extra PIWI domains. MdAGO4 had two sets of PAZ, MID, and PIWI domains which repeated in direct tandem and a gamma-thionin was located before these domains. Gamma-thionins C-termini domain is an important determinant on antifungal activity and antimicrobial activity. These peptides were named gamma-thionins or defensins that can be classified into four main subtypes according to their specific functions. Gamma-thionins are small cationic peptides with different and special abilities. They are able to inhibit digestive enzymes or act against bacteria and/or fungi [31]. Extra domains may be related to duplication in loci LuAGO1 and MdAGO5 but existence of gamma-thionin domains in the Argonaute protein is not clear and needs more investigation. MdAGO5 in addition to PAZ, MID, and PIWI domains had two Argonaute unusual domains Zinc finger C-x8-C-x5-C-x3-H type and ab-hydrolase associated lipase. Zinc finger proteins belong to a superfamily divided into nine classes (C2H2, C8, C6, C3HC4, C2HC, C2HC5, C4, C4HC3, and CCCH) according to the numbers of conserved cysteine (C) and histidine (H) residues and the spacing between these conserved residues [32]. The CCCH-type zinc finger genes are widely present in eukaryotes. Most of the characterized CCCH-type zinc finger proteins are associated with RNA metabolism, including RNA cleavage, RNA degradation, RNA polyadenylation, or RNA export by binding to RNA [33]. In Arabidopsis, the CCCH-type protein HUA1 is involved in the processing of AGAMOUS pre-mRNA as an RNA-binding protein during flower development. Another Arabidopsis CCCH-type protein, AtTZF1, shuttling between the nucleus and cytoplasmic foci, can bind both DNA and RNA in vitro and is likely involved in gibberellin acid/abscisic acid-mediated developmental and environmental responses through DNA or RNA regulation [34]. CCCH-type gene family may be involved in abiotic or biotic stress tolerance like plant-pathogen interaction, which regulates resistance to the fungal pathogen, enhancing tobacco tolerance to salt stress. Most of the characterized CCCH-type zinc finger proteins are associated with RNA metabolism by binding to the target mRNA and transcriptionally regulate gene expression by binding to DNA [35]. C3H12 may regulate disease resistance by promoting the cleavage or degradation of mRNAs of some defense-responsive genes that encoded proteins function as negative regulators in rice-Xoo interaction and thus remove the suppression on defense positive regulators [32, 35].

MdAGO13 in addition to PAZ, MID, and PIWI domains had two domains ribosome-inactivating proteins (RIPs) and DYW family of nucleic acid deaminases. Ribosome-inactivating proteins (RIPs) are toxic-glycosidases that depurinate the universally conserved alpha-sarcin loop of large rRNAs. This depurination inactivates the ribosome, thereby blocking its further participation in protein synthesis. RIPs are widely distributed among different plant genera and within a variety of different tissues [36]. Recent work has shown that enzymatic activity of at least some RIPs is not limited to site-specific action on the large rRNAs of ribosomes but extends to depurination and even nucleic acid scission of other targets. For plants, RIPs have been linked to defense by antiviral, antifungal, and insecticidal properties demonstrated in vitro and in transgenic plants [37]. DYW family of nucleic acid deaminases is a family of nucleic acid deaminases prototyped by the plant PPR DYW proteins that are implicated in chloroplast and mitochondrial RNA transcript maturation by numerous C to U editing events. The name derives from the DYW motif present at the C-terminus of the classical plant PPR DYW deaminases. Members of this family are present in bacteria, plants, Naegleria, and fungi [38]. Plants and Naegleria show lineage-specific expansions of this family. The classical DYW family contains an additional C-terminal metal-binding cluster composed of 2 histidines and a CxC motif and is often fused to PPR repeats. Ascomycete versions, which are independent lateral transfers, contain a large insert within the domain and are often fused to ankyrin repeats. Bacterial versions are predicted to function as toxins in polymorphic toxin systems [39].

FvAGO1 had alpha-crystallin domain (ACD) of alpha-crystallin-type small (s) heat shock proteins (*Hsps*) placed in front of the PAZ domain. Alpha-crystallin domain (ACD) of alpha-crystallin-type small (s) heat shock proteins (sHsps) is small stress induced proteins with monomeric masses between 12 and 43 kDa, whose common feature is the alpha-crystallin domain (ACD). sHsps are generally active as large oligomers consisting of multiple subunits and are believed to be ATP-independent chaperones that prevent aggregation and are important in refolding in combination with other Hsps. *α*-Crystallins were originally recognized as proteins contributing to the transparency of the mammalian eye lens. Subsequently, they have been found in many, but not all, members of the archaea, bacteria, and Eucarya [40]. Since *α*-crystallins are induced by a temperature upshift in many organisms, they are often referred to as small heat shock proteins (sHsps) or, more accurately, *α*-Hsps. *α*-Crystallins are integrated into a highly flexible and synergistic multichaperone network evolved to secure protein quality control in the cell. Their chaperone activity is limited to the binding of unfolding intermediates in order to protect them from irreversible aggregation [41].

FvAGO9 had regular domains and additionally GT1-SUCORUS SYNTAS domain which is located after PIWI. Glycosyltransferases catalyze the transfer of sugar moieties from activated donor molecules to specific acceptor molecules, forming glycosidic bonds. This family is most closely related to the GT1 family of glycosyltransferases. Glycosyltransferases are a ubiquitous group of enzymes that catalyse the transfer of a sugar moiety from an activated sugar donor onto saccharide or nonsaccharide acceptors. The sucrose-phosphate synthases in this family may be unique to plants and photosynthetic bacteria. This enzyme catalyzes the synthesis of sucrose 6-phosphate from fructose 6-phosphate and uridine 5′-diphosphate-glucose, a key regulatory step of sucrose metabolism. The activity of this enzyme is regulated by phosphorylation and moderated by the concentration of various metabolites and light [42]. These enzymes are present in both prokaryotes and eukaryotes, and they generally display exquisite specificity for both the glycosyl donor and the acceptor substrates. In eukaryotes, most of the glycosylation reactions that generate the diversity of oligosaccharide structures of eukaryotic cells occur in the Golgi apparatus [43].

Sequence of BrAGO5 in addition to PAZ, MID, and PIWI domains also has two cims N terminal like domain which are located after the PIWI domain, CIMS: Cobalamin-independent methonine synthase, or MetE, C-terminal domain like. Many members have been characterized as 5-methyltetrahydropteroyltriglutamate-homocysteine methyltransferases, mostly from bacteria and plants. This enzyme catalyses the last step in the production of methionine by transferring a methyl group from 5-methyltetrahydrofolate to L-homocysteine without using an intermediate methyl carrier [44]. The active enzyme has a dual (beta-alpha) 8-barrel structure, and this model covers the C-terminal barrel and a few single-barrel sequences most similar to the C-terminal barrel. It is assumed that the homologous N-terminal barrel has evolved from the C-terminus via gene duplication and has subsequently lost binding sites, and it seems as if the two barrels forming the active enzyme may sometimes reside on different polypeptides. The C-terminal domain incorporates the zinc ion, which binds and activates homocysteine. Side chains from both barrels contribute to the binding of the folate substrate [44]. This is the first report of unusual Argonaute domain that needs more experimental analysis to find the role of these domains especially for Argonaute genes.

The results of number of alternative transcripts related to AGO gene analysis show that some of the plants do not have alternative transcripts of Argonaute gene but some of the plant such us* Aquilegia coerulea* most of the Argonaute loci produce alternative transcripts and the level of these alternative transcripts was highest in comparison with loci in other studied plants. Chromosome location of the Argonaute gene map for available* A. thaliana, Brachypodium distachyon*,* Cucumis sativus*,* Glycine max*,* Medicago truncatula*,* Populus trichocarpa,* and* Vitis vinifera* map chromosomes proves they do not have synteny. Expression profiles of Argonaute proteins in some of the plants indicated that these proteins have high expression in the seed, leaf, root, and shoot in the studied plant. Previous study data demonstrated that these genes exhibited different expression levels in biotic and abiotic stress treatments such as response to cold, salt and dehydration stress, water deficit, and virus infection stresses. This shows that the transcriptional and posttranscriptional control of gene expression mediated by sRNAs are probably involved in plant adaptation to biotic and abiotic environmental Changes. Argonaute expression in the different tissue and in different circumstance may show the probable roles of these genes in plant growth and development. [12, 18–20].

## 5. Conclusion

This study provides a comparative genomic analysis addressing the phylogenetic relationships and evolution of the Argonaute gene family in 32 plant species from different families. The results of this study demonstrate that Argonaute proteins in the phylogenetic analysis have three highly conserved subfamilies existing in plants. Existence of PAZ, MID, and PIWI domain in all of the sequences revealed that this protein has high conservation in different plant species. However, the role and function of some unusual domains are not clear. Future studies using these Argonautes will help us to determine the biological function of these genes. Expression of Argonaute proteins in all of the tissue showed that this protein was involved in most pathways of the plant system. Numbers of alternative transcripts relevant to Argonaute gene in the plant were very diverse. Some of the plants such us Aquilegia coerulea have alternative transcripts and the level of these alternative transcripts was highest in comparison with other plants.

## Supplementary Material

Biochemical characters of 437 Argonaute sequences related to *Arabidopsis thaliana* and 32 plants which these sequences obtained from phytozome database v9.1 (http://www.phytozome.net/). Each sequence was named base on first letter of plant name. Gene ID for each sequences proper to phytozome database. Length and weight of each sequence was presented in number of amino acid (aa) and kilodaltons (kD) respectively.

## Figures and Tables

**Table 1:** Genomic characteristic and number of the Argonaute proteins and levels of alternative transcripts in the plants were studied.

Family	Species	Number of chromosome	Genome size	Transcripts	Loci	Database	Number of Argonaute	Number of alternative transcripts related to AGO protein
Fabidae	*Manihot esculenta *	18	533	34,151	30,666		13	AGO9 (2)
	*Ricinus communis *	10	400	31221	—		9	
	*Linum usitatissimum *	15	318.3	43,484	26,374		18	
	*Populus trichocarpa *	19	403	45033	40668		15	
	*Medicago truncatula *	8	241	53423	50962		9	AGO5 (3)
	*Phaseolus vulgaris *	11	486.9	—	26,374		14	AGO4 (2), AGO7 (2), AGO11 (2), AGO13 (2)
	*Glycine max *	20	975	—	66,153		21	AGO2 (2), AGO3 (3), AGO4 (2), AGO11 (2), AGO13 (2), AGO15 (2), AGO18 (4), AGO19 (3), AGO20 (2)
	*Cucumis sativus *	7	203	32528	21491		7	AGO1 (2), AGO2 (4), AGO3 (5), AGO5 (3), AGO6 (3)
	*Prunus persica *	8	227.3	28702	27864		11	AGO7 (2)
	*Malus domestica *	17	881.3	63,541	26,374		15	
	*Fragaria vesca *	7	240	32,831	32,831		12	
Malvidae	*Arabidopsis thaliana *	5	135	35386	27416		10	AGO4 (2), AGO1 (3), AGO10 (2)
	*Arabidopsis lyrata *	8	207	32670	—		10	
	*Capsella rubella *	8	134.8	—	26,521		10	
	*Brassica rapa *	10	283.8	63,541	26,374		13	
	*Thellungiella halophila *	7	243.1		26,351		9	AGO3 (3)
	*Carica papaya *	9	135	27796	27332	JGI	6	
	*Gossypium raimondii *	13	880	—	—		13	AGO1 (2), AGO3 (2), AGO4 (2), AGO5 (8), AGO8 (3), AGO9 (7), AGO10 (3), AGO11 (6), AGO12 (2), AGO13 (5),
	*Theobroma cacao *	10	346	—	29,452		11	AGO1 (5), AGO3 (3), AGO6 (2), AGO7 (2), AGO9 (4), AGO10 (6)
	*Citrus sinensis *	9	319	46,147	25,376		10	AGO4 (4), AGO5 (2), AGO7 (5), AGO9 (7)
	*Citrus clementina *	9	296	35,976	25,385		15	AGO6 (2), AGO14 (3),
	*Eucalyptus grandis *	11	691		36,376		14	AGO1 (3), AGO9 (3), AGO11 (3), AGO14 (2),
Other plants	*Vitis vinifera *	19	487	26346	26346		15	
	*Solanum tuberosum *	12	800	51470	35,119		11	AGO1 (2), AGO2 (2), AGO3 (4), AGO4 (3), AGO6 (2), AGO8 (2),
	*Solanum lycopersicum *	12	900	34,727	34,727		15	
	*Mimulus guttatus *	7	321	28282	26718		13	
	*Aquilegia coerulea *	7	302	41063	24,823		18	AGO1 (4), AGO2 (6), AGO3 (6), AGO4 (4), AGO7 (2), AGO8 (4), AGO9 (5), AGO10 (3), AGO11 (7), AGO13 (4), AGO15 (17), AGO16 (7), AGO17 (4), AGO18 (11)
Grass	*Sorghum bicolor *	10	698	36338	34,496		14	AGO7 (2)
	*Zea mays *	10	2500	—	—		16	AGO2 (3), AGO3 (3), AGO6 (2), AGO7 (2), AGO16 (3)
	*Setaria italica *	9	405.7	40599	35,471		15	AGO9 (2), AGO10 (2)
	*Panicum virgatum *	7	1,358	—	4,193		24	AGO7 (2), AGO8 (2)
	*Oryza sativa *	12	372	66338	55,986		15	AGO2 (2), AGO3 (2), AGO5 (4), AGO12 (2), AGO14 (2), AGO3 (2),
	*Brachypodium distachyon *	5	272	31029	26,552		16	AGO11 (2), AGO12 (3)

**Table 2:** The expression profile for *Arabidopsis thaliana, Oryza sativa*, *Medicago truncatula, Vitis vinifera, Glycine max*, *Populus trichocarpa, Prunus persica, Malus domestica, and Aquilegia coerulea* Argonaute genes from NCBI EST database. The black points indicate the expression data for Argonaute genes, and the blank shows that no expression could be detected. The names of expressed genes are shown on the left side of the table; genes with no expression data are not shown.

Gene Name	Root	Leaves	Shoot	Flower	Seed	Stem	Hypocotyl	Pollen	Panicle	Callus	Pistil	Ovule	Pericarp	Cotyledons	Cambium	Buds	Mesocarp	Fruit
AtAGO1	●	●		●	●		●					●						
AtAGO2	●	●		●	●							●						
AtAGO3												●						
AtAGO4					●													
AtAGO5					●													
AtAGO6				●	●											●		
AtAGO7												●						

OsAGO1	●	●	●		●			●	●	●	●							
OsAGO2	●			●	●				●	●								
OsAGO3	●	●	●	●	●				●	●	●							
OsAGO4			●	●						●								
OsAGO5		●			●					●								

MtAGO1	●	●		●	●	●								●				
MtAGO2					●	●												
MtAGO3	●	●		●	●	●								●				
MtAGO4	●			●										●				
MtAGO5	●				●													
MtAGO6	●									●								

VvAGO1					●								●					

GmAGO1	●	●	●	●	●													
GmAGO2		●			●					●				●				
GmAGO3	●	●	●	●	●		●			●				●				
GmAGO4		●			●													
GmAGO5			●		●									●				
GmAGO6					●					●				●				

PtAGO1	●	●				●									●			
PtAGO2	●	●				●										●		
PtAGO3	●	●				●										●		
PtAGO4	●	●				●									●	●		
PtAGO5		●				●									●			

PprAGO1																	●	●

MdAGO1		●		●												●		●
MdAGO2																●		●
MdAGO3		●																●

AcAGO1	●	●	●	●														
AcAGO2	●	●	●	●														
AcAGO3	●	●	●	●														
AcAGO4	●	●	●	●														
AcAGO5	●	●	●	●														
AcAGO6	●	●	●	●														
AcAGO7	●	●	●	●														
AcAGO8	●	●	●	●														
AcAGO9	●	●	●	●														
AcAGO10	●	●	●	●														
AcAGO11	●	●	●	●														
AcAGO12	●	●	●	●														
