# Supplementary material for: In Silico Identification, Phylogenetic and Bioinformatic Analysis of Argonaute Genes in Plants
Source: Int J Genomics. 2014 Sep 15;2014:967461. doi: 10.1155/2014/967461 (PMC4181786; doi:10.1155/2014/967461)
Supplement: Supplementary file 1 — Biochemical characters of 437 Argonaute sequences related to Arabidopsis thaliana and 32 plants which these sequences obtained from phytozome database v9.1 (http://www.phytozome.net/). Each sequence was named base on first letter of plant name. Gene ID for each sequences proper to phytozome database. Length and weight of each sequence was presented in number of amino acid (aa) and kilodaltons (kD) respectively. [file 967461.f1.pdf]

Table 3. Biochemical characters of all Argonaute sequences

Name	Gene ID	Length	Weight	Isoelectric point	Aliphatic index	Hydrophobic	Hydrophilic	Negatively Charged	Positively Charged	Alpha helix	Beta strand
Sl-AGO1	Solyc06g073540.2.1	913	101.879	9.21	81.654	0.479	0.265	0.108	0.125	18	43
Sl-AGO2	Solyc06g073530.1	904	101.278	9.2	83.219	0.468	0.274	0.105	0.124	21	39
Sl-AGO3	Solyc06g074730.2	1011	112.64	9.46	76.439	0.481	0.262	0.096	0.127	23	49
Sl-AGO4	Solyc06g072300.2	1054	116.855	9.58	74.725	0.488	0.267	0.094	0.127	30	33
Sl-AGO5	Solyc02g069270.2	977	109.978	9.3	71.699	0.477	0.267	0.104	0.129	25	38
Sl-AGO6	Solyc02g069280.2	999	112.671	9.06	77.728	0.446	0.273	0.121	0.138	26	39
Sl-AGO7	Solyc02g069260.2	1042	115.999	9.55	73.868	0.488	0.262	0.097	0.131	24	32
Sl-AGO8	Solyc03g111760.2	916	104.683	9.78	86.91	0.471	0.287	0.084	0.130	20	50
Sl-AGO9	Solyc03g098280.2	980	109.211	9.38	79.48	0.477	0.273	0.099	0.126	29	30
Sl-AGO10	Solyc07g049500.2	903	101.552	8.94	86.866	0.463	0.261	0.115	0.127	29	35
Sl-AGO11	Solyc01g010970.2	1000	114.006	9.42	86.43	0.450	0.291	0.095	0.127	32	38
Sl-AGO12	Solyc01g096750.1	881	98.778	9.43	81.555	0.465	0.276	0.101	0.129	25	32
Sl-AGO13	Solyc01g008960.2	909	101.751	9.18	81.232	0.475	0.268	0.108	0.125	18	46
Sl-AGO14	Solyc12g006790.1.1	933	105.493	9.43	86.088	0.459	0.282	0.100	0.135	33	40
Sl-AGO15	Solyc09g082830.2.1	982	110.521	9.45	80.295	0.462	0.275	0.101	0.135	25	38
Mg-AGO1	mgv1a001065m	898	99.844	8.99	87.149	0.465	0.286	0.104	0.118	23	44
Mg-AGO2	mgv1a022215m	1008	112.812	9.61	79.286	0.477	0.274	0.093	0.131	26	50
Mg-AGO3	mgv1a000944m	936	104.007	9.0	79.957	0.476	0.277	0.109	0.122	30	37
Mg-AGO4	mgv1a000822m	971	108.361	9.53	81.03	0.479	0.269	0.094	0.130	30	33
Mg-AGO5	mgv1a002480m	668	74.61	9.42	89.88	0.503	0.240	0.102	0.136	20	22
Mg-AGO6	mgv1a001217m	863	97.031	9.94	85.724	0.484	0.267	0.086	0.144	22	41
Mg-AGO7	mgv1a001669m	776	87.709	6.38	90.374	0.455	0.281	0.128	0.119	18	33
Mg-AGO8	mgv1a000922m	942	105.26	9.33	79.575	0.485	0.256	0.103	0.132	23	34
Mg-AGO9	mgv1a001365m	833	95.127	9.2	76.723	0.448	0.288	0.100	0.126	25	34
Mg-AGO10	mgv1a000545m	1084	120.856	9.45	71.587	0.478	0.275	0.094	0.123	30	32

Mg-AGO11	mgv1a001084m	893	99.234	9.55	83.919	0.496	0.259	0.095	0.127	16	38
Mg-AGO12	mgv1a000751m	995	111.997	9.53	74.693	0.464	0.275	0.104	0.137	24	39
Mg-AGO13	mgv1a000885m	951	108.069	9.24	83.775	0.461	0.263	0.114	0.138	28	47
Rc-AGO1	Rcommunis|27389	863	97.113	9.41	82.897	0.459	0.286	0.101	0.132	23	33
Rc-AGO2	Rcommunis|29589	987	110.115	9.63	78.034	0.467	0.278	0.097	0.137	31	35
Rc-AGO3	Rcommunis|29677	1063	117.809	9.49	73.17	0.486	0.271	0.094	0.125	26	33
Rc-AGO4	Rcommunis|29684	921	102.866	9.2	79.848	0.477	0.264	0.109	0.127	16	42
Rc-AGO5	Rcommunis|29807	944	107.108	9.19	85.604	0.458	0.280	0.107	0.131	33	39
Rc-AGO6	Rcommunis|29813	1020	116.266	9.62	81.716	0.459	0.284	0.093	0.133	38	23
Rc-AGO7	Rcommunis|29828	917	102.702	9.11	81.167	0.480	0.264	0.108	0.123	25	38
Rc-AGO8	Rcommunis|29844	986	110.938	9.42	79.696	0.462	0.277	0.100	0.133	37	30
Rc-AGO9	Rcommunis|30093	972	107.961	9.14	78.117	0.473	0.278	0.102	0.120	27	30
Fv-AGO1	mrna00394.1-v1.0-hybrid	1079	120.329	9.32	81.437	0.467	0.286	0.103	0.125	28	47
Fv-AGO2	mrna16928.1-v1.0-hybrid	1080	120.083	9.41	85.917	0.483	0.265	0.103	0.130	24	50
Fv-AGO3	mrna28930.1-v1.0-hybrid	1007	112.714	9.46	80.933	0.473	0.271	0.098	0.133	32	39
Fv-AGO4	mrna06591.1-v1.0-hybrid	888	100.229	9.63	80.518	0.476	0.273	0.095	0.135	24	38
Fv-AGO5	mrna16926.1-v1.0-hybrid	845	94.679	8.89	86.331	0.483	0.254	0.115	0.127	19	40
Fv-AGO6	mrna01157.1-v1.0-hybrid	1065	118.687	9.46	87.005	0.485	0.270	0.099	0.128	24	51
Fv-AGO7	mrna07657.1-v1.0-hybrid	975	108.259	8.92	83.159	0.475	0.272	0.111	0.122	22	40
Fv-AGO8	mrna09291.1-v1.0-hybrid	1090	120.438	9.42	74.092	0.477	0.272	0.094	0.122	33	29
Fv-AGO9	mrna09290.1-v1.0-hybrid	1956	218.156	9.03	77.515	0.489	0.265	0.104	0.119	62	65
Fv-AGO10	mrna14489.1-v1.0-hybrid	853	94.968	9.43	80.551	0.468	0.274	0.101	0.127	26	31
Fv-AGO11	mrna14490.1-v1.0-hybrid	1070	119.767	9.95	77.551	0.466	0.271	0.093	0.145	30	37
Fv-AGO12	mrna20067.1-v1.0-hybrid	1063	117.875	9.44	74.516	0.483	0.270	0.102	0.128	29	36
Tc-AGO1	Thecc1EG042027t1	913	101.897	9.2	81.512	0.478	0.272	0.104	0.123	22	41
Tc-AGO2	Thecc1EG041547t1	988	109.667	9.7	73.887	0.455	0.291	0.092	0.136	28	40
Tc-AGO3	Thecc1EG037973t1	943	106.727	9.36	83.627	0.458	0.276	0.104	0.134	32	42

Tc-AGO4	Thecc1EG001025t1	970	107.939	9.25	77.67	0.461	0.276	0.105	0.131	28	43
Tc-AGO5	Thecc1EG001023t1	978	109.528	9.41	78.916	0.459	0.283	0.101	0.133	23	47
Tc-AGO6	Thecc1EG002192t1	903	100.8	9.51	88.062	0.476	0.264	0.102	0.135	19	38
Tc-AGO7	Thecc1EG029345t1	1016	113.818	9.51	78.248	0.491	0.238	0.103	0.133	22	46
Tc-AGO8	Thecc1EG029341t1	904	100	9.39	83.992	0.472	0.247	0.092	0.118	21	45
Tc-AGO9	Thecc1EG013468t1	1063	117.088	9.51	74.647	0.488	0.272	0.090	0.124	26	37
Tc-AGO10	Thecc1EG020717t1	994	111.528	9.43	78.179	0.471	0.270	0.100	0.132	34	30
Tc-AGO11	Thecc1EG011631t1	1014	115.046	9.54	80.838	0.461	0.284	0.094	0.129	33	35
Sb-AGO1	Sb01g004920.1	1067	117.271	9.54	74.658	0.503	0.248	0.093	0.126	23	47
Sb-AGO2	Sb01g011870.1	1255	135.416	9.85	72.151	0.519	0.268	0.074	0.116	28	45
Sb-AGO3	Sb01g011880.1	1087	119.281	9.49	78.721	0.498	0.247	0.094	0.129	26	44
Sb-AGO4	Sb01g032060.1	1033	115.846	9.55	83.911	0.489	0.245	0.097	0.132	37	33
Sb-AGO5	Sb02g005150.1	1036	112.033	9.67	76.448	0.527	0.250	0.080	0.118	20	46
Sb-AGO6	Sb02g032980.1	1044	114.154	9.51	71.619	0.509	0.239	0.098	0.132	26	43
Sb-AGO7	Sb03g011020.1	900	100.671	9.29	81.744	0.469	0.268	0.107	0.130	23	41
Sb-AGO8	Sb04g038420.1	1028	113.75	9.55	76.77	0.500	0.246	0.097	0.129	29	34
Sb-AGO9	Sb06g025560.1	1082	119.861	9.6	74.667	0.490	0.265	0.091	0.125	25	40
Sb-AGO10	Sb06g028510.1	1092	116.914	9.55	73.819	0.516	0.245	0.091	0.125	23	38
Sb-AGO11	Sb09g000530.1	1109	122.108	9.64	72.777	0.485	0.270	0.088	0.123	25	37
Sb-AGO12	Sb09g030910.1	909	101.474	9.07	76.975	0.475	0.263	0.111	0.128	24	35
Sb-AGO13	Sb10g023230.1	975	108.899	9.48	81.108	0.489	0.256	0.095	0.129	26	41
Sb-AGO14	Sb10g031030.1	1016	112.888	9.16	78.72	0.484	0.261	0.102	0.122	35	26
Pv-AGO1	Phvul.003G160200.1	376	42.584	9.27	99.282	0.489	0.266	0.093	0.128	12	25
Pv-AGO2	Phvul.003G046700.1	1027	117.582	9.39	82.016	0.440	0.290	0.097	0.128	33	31
Pv-AGO3	Phvul.003G160000.1	906	102.691	9.22	89.812	0.466	0.266	0.108	0.134	27	47
Pv-AGO4	Phvul.009G199500.1	908	103.144	9.28	84.251	0.457	0.280	0.105	0.132	32	45
Pv-AGO5	Phvul.011G169400.1	886	99.248	8.96	88.409	0.465	0.277	0.108	0.123	23	44
Pv-AGO6	Phvul.011G088200.1	995	109.725	9.71	79.487	0.481	0.278	0.089	0.125	27	39

Pv-AGO7	Phvul.008G206600.1	907	101.348	9.29	83.55	0.472	0.269	0.109	0.130	19	39
Pv-AGO8	Phvul.008G206500.1	904	101.388	9.34	83.186	0.467	0.271	0.110	0.133	18	34
Pv-AGO9	Phvul.004G142900.1	1063	117.405	9.46	73.735	0.494	0.263	0.093	0.124	25	37
Pv-AGO10	Phvul.007G062800.1	974	109.675	9.41	78.573	0.464	0.271	0.104	0.137	34	33
Pv-AGO11	Phvul.007G278600.1	974	109.675	9.41	78.573	0.464	0.271	0.104	0.137	34	38
Pv-AGO12	Phvul.006G021200.1	919	103.112	9.11	81.306	0.479	0.256	0.113	0.129	18	36
Pv-AGO13	Phvul.006G131700.1	979	111.161	9.28	79.714	0.469	0.266	0.106	0.134	21	43
Pv-AGO14	Phvul.002G100100.1	971	109.753	9.24	76.262	0.457	0.263	0.113	0.138	24	42
Al-AGO1	481584	924	103.089	9.16	80.487	0.474	0.275	0.106	0.123	26	36
Al-AGO2	473645	1023	113.989	9.54	67.986	0.482	0.241	0.114	0.144	29	43
Al-AGO3	476072	989	113.133	9.38	82.174	0.446	0.287	0.100	0.131	32	30
Al-AGO4	326484	848	95.299	9.41	81.91	0.467	0.279	0.098	0.126	21	40
Al-AGO5	913356	1110	122.476	9.72	70.477	0.463	0.274	0.104	0.141	27	55
Al-AGO6	933874	877	98.734	9.17	83.9	0.469	0.267	0.108	0.130	19	39
Al-AGO7	489025	902	100.895	9.38	80.022	0.475	0.286	0.098	0.123	22	42
Al-AGO8	481718	1001	111.224	9.67	78.771	0.454	0.296	0.095	0.132	30	36
Al-AGO9	473977	1052	116.545	9.51	74.772	0.476	0.278	0.094	0.126	28	34
Al-AGO10	948772	983	110.411	9.5	79.919	0.464	0.278	0.099	0.135	32	37
Csi-AGO1	orange1.1g002636m	898	100.949	9.6	77.717	0.470	0.280	0.092	0.126	16	37
Csi-AGO2	orange1.1g003630m	806	91.162	9.1	88.511	0.485	0.283	0.097	0.113	22	39
Csi-AGO3	orange1.1g001684m	1030	117.035	9.37	81.291	0.450	0.295	0.094	0.125	34	31
Csi-AGO4	orange1.1g001954m.1	992	111.515	9.49	79.496	0.459	0.282	0.098	0.133	36	31
Csi-AGO5	orange1.1g002661m.1	895	100.498	9.57	86.458	0.473	0.272	0.099	0.132	20	35
Csi-AGO6	orange1.1g048669m	568	64.051	9.94	83.398	0.470	0.271	0.095	0.143	15	22
Csi-AGO7	orange1.1g002449m.1	920	102.996	9.17	83.12	0.473	0.267	0.111	0.128	21	38
Csi-AGO8	orange1.1g036169m	619	69.384	9.86	85.202	0.465	0.271	0.092	0.142	22	25
Csi-AGO9	orange1.1g001466m.1	1073	118.337	9.47	72.022	0.482	0.272	0.093	0.122	24	38
Csi-AGO10	orange1.1g002204m	954	106.772	9.33	82.369	0.477	0.286	0.095	0.118	26	41

Zm-AGO1	GRMZM2G441583_T01	1102	121.056	9.63	76.243	0.502	0.259	0.086	0.119	28	39
Zm-AGO2	GRMZM2G141818_T03.1	910	101.543	9.04	78.176	0.473	0.264	0.112	0.127	23	33
Zm-AGO3	GRMZM2G079080_T02.1	966	108.385	9.47	83.282	0.487	0.257	0.099	0.133	32	32
Zm-AGO4	AC189879.3_FGT003	984	109.604	9.39	83.262	0.502	0.251	0.093	0.126	26	38
Zm-AGO5	GRMZM2G105250_T01	1053	115.706	9.38	70.684	0.499	0.242	0.105	0.135	30	36
Zm-AGO6	GRMZM2G007791_T01.1	1033	112.154	9.54	77.173	0.501	0.256	0.094	0.129	28	32
Zm-AGO7	GRMZM2G059033_T01.1	1013	110.399	9.49	78.569	0.515	0.242	0.093	0.124	23	38
Zm-AGO8	GRMZM2G039455_T01	1078	119.683	9.54	75.584	0.488	0.268	0.092	0.122	26	40
Zm-AGO9	GRMZM2G589579_T01	898	100.533	9.35	82.149	0.474	0.258	0.109	0.134	23	40
Zm-AGO10	GRMZM5G892991_T01	1032	115.298	9.56	84.176	0.494	0.236	0.098	0.132	31	33
Zm-AGO11	GRMZM2G457370_T01.1	919	103	8.65	86.35	0.468	0.247	0.129	0.137	28	38
Zm-AGO12	GRMZM2G354867_T01	1039	113	9.43	77.584	0.500	0.257	0.095	0.126	21	44
Zm-AGO13	AC209206.3_FGT011	1092	121	9.35	72.747	0.483	0.267	0.097	0.120	28	37
Zm-AGO14	GRMZM2G361518_T01.2	1027	114	9.41	78.257	0.484	0.260	0.100	0.128	33	27
Zm-AGO15	GRMZM2G123063_T01	941	103	9.41	75.143	0.505	0.240	0.096	0.123	18	44
Zm-AGO16	GRMZM2G461936_T02.1	869	95.04	9.45	78.44	0.494	0.268	0.089	0.124	20	35
Lu-AGO1	Lus10014386	1113	124	9.46	80.045	0.470	0.279	0.098	0.127	29	50
Lu-AGO2	Lus10025537	922	102	8.94	91.95	0.500	0.248	0.108	0.120	24	36
Lu-AGO3	Lus10006627	1007	112	9.45	77.09	0.467	0.279	0.097	0.130	34	32
Lu-AGO4	Lus10017983	1186	128	9.59	68.7	0.500	0.277	0.083	0.115	23	38
Lu-AGO5	Lus10015155	884	98.7	9.45	82.7	0.484	0.267	0.10	0.129	21	38
Lu-AGO6	Lus10031331	1094	120	9.53	71.08	0.476	0.282	0.093	0.126	26	34
Lu-AGO7	Lus10037136	1021	115	9.43	79.87	0.45	0.289	0.092	0.124	26	50
Lu-AGO8	Lus10041978	1178	129	9.58	72.95	0.497	0.277	0.083	0.117	23	43
Lu-AGO9	Lus10040619	1173	131	9.13	77.83	0.470	0.257	0.115	0.13	29	55
Lu-AGO10	Lus10029989	919	103	9.57	85	0.473	0.277	0.092	0.127	26	35
Lu-AGO11	Lus10023882	983	109	8.24	80.02	0.46	0.307	0.104	0.107	25	55
Lu-AGO12	Lus10035331	913	101	9.31	86.78	0.476	0.269	0.101	0.125	25	38

Lu-AGO13	Lus10036794	1020	115	9.41	78.147	0.449	0.294	0.091	0.123	24	50
Lu-AGO14	Lus10036795	875	98.826	9.37	84.503	0.464	0.278	0.098	0.129	22	45
Lu-AGO15	Lus10039386	874	98.4	9.2	84.00	0.478	0.26	0.104	0.129	31	30
Lu-AGO16	Lus10026750	873	97.	9.22	84.834	0.491	0.253	0.108	0.127	22	34
Lu-AGO17	Lus10018290	1021	114	9.33	73.27	0.476	0.250	0.112	0.135	25	46
Lu-AGO18	Lus10031904	1053	116	9.63	72.65	0.477	0.282	0.091	0.128	24	35
Mt-AGO1	Medtr3g083300.1	947	105	8.83	87.38	0.479	0.272	0.108	0.117	30	31
Mt-AGO2	Medtr3g010650.1	876	99.92	9.35	84.11	0.436	0.298	0.099	0.131	23	37
Mt-AGO3	Medtr2g028910.1	1038	117	9.13	77.9	0.457	0.269	0.116	0.135	23	47
Mt-AGO4	Medtr5g087890.1	929	104	9.36	87.33	0.473	0.270	0.104	0.132	21	45
Mt-AGO5	Medtr5g087870.1	948	106	9.27	83	0.474	0.274	0.102	0.123	20	43
Mt-AGO6	Medtr5g042590.1	1016	116	9.32	81.2	0.432	0.310	0.096	0.125	36	36
Mt-AGO7	Medtr1g106830.1	902	102	9.48	80	0.461	0.277	0.104	0.136	23	36
Mt-AGO8	Medtr4g113200.1	876	98	9.04	86.6	0.463	0.274	0.106	0.128	23	42
Mt-AGO9	Medtr4g083610.1	977	109.82	9.43	76.18	0.467	0.278	0.097	0.128	22	41
Cr-AGO1	Carubv10008180m	1021	113.716	9.71	71.577	0.478	0.245	0.109	0.146	29	42
Cr-AGO2	Carubv10008158m	1068	118.232	9.5	73.567	0.477	0.281	0.092	0.124	28	31
Cr-AGO3	Carubv10010881m	948	105.973	9.32	70.622	0.467	0.267	0.112	0.131	24	42
Cr-AGO4	Carubv10022614m	877	98.706	9.16	84.47	0.471	0.270	0.107	0.128	20	39
Cr-AGO5	Carubv10025461m	1023	113.343	9.76	78.387	0.471	0.276	0.096	0.136	30	40
Cr-AGO6	Carubv10022599m	923	102.816	9.25	80.585	0.481	0.271	0.103	0.124	25	40
Cr-AGO7	Carubv10019715m	989	113.448	9.45	82.073	0.446	0.277	0.101	0.135	35	29
Cr-AGO8	Carubv10002706m	861	96.958	9.5	81.15	0.467	0.280	0.098	0.130	25	31
Cr-AGO9	Carubv10000166m	922	103.174	9.39	81.475	0.479	0.277	0.101	0.127	24	42
Cr-AGO10	Carubv10025804m	1018	114.413	9.61	80.147	0.464	0.276	0.097	0.139	36	37
Eg-AGO1	Eucgr.B03780.1	918	102.314	9.29	83.399	0.485	0.260	0.107	0.127	19	44
Eg-AGO2	Eucgr.B03907.1	1004	110.688	9.6	79.293	0.503	0.238	0.101	0.134	32	35

Eg-AGO3	Eucgr.B03909.1	1000	110.911	9.5	79.98	0.495	0.247	0.103	0.133	29	36
Eg-AGO4	Eucgr.D00105.1	891	101.972	9.28	84.77	0.464	0.275	0.102	0.130	20	40
Eg-AGO5	Eucgr.D00106.1	955	109	9.22	75	0.465	0.277	0.098	0.137	24	35
Eg-AGO6	Eucgr.D00109.1	958	109.03	8.82	75.908	0.442	0.271	0.121	0.134	30	32
Eg-AGO7	Eucgr.D01097.1	968	111.83	9.34	79.215	0.441	0.259	0.118	0.147	32	36
Eg-AGO8	Eucgr.G02476.1	1016	114.972	9.49	82.687	0.460	0.273	0.100	0.135	25	33
Eg-AGO9	Eucgr.H00532.1	980	110.044	9.54	79.786	0.465	0.277	0.098	0.137	39	28
Eg-AGO10	Eucgr.H00615.1	1051	116.566	9.45	72.997	0.459	0.281	0.102	0.133	24	37
Eg-AGO11	Eucgr.J00634.1	949	105.807	9.13	81.834	0.476	0.277	0.102	0.120	20	40
Eg-AGO12	Eucgr.K00735.1	904	101.337	9.36	84.602	0.465	0.279	0.105	0.133	23	34
Eg-AGO13	Eucgr.K00736.1	880	98.879.38	9.38	85.045	0.459	0.277	0.106	0.134	23	33
Eg-AGO14	Eucgr.K02304.1	1074	119.402	9.43	70.168	0.478	0.265	0.098	0.128	25	33
Si-AGO1	Si004216m	849	94.87	9.39	87.797	0.459	0.278	0.104	0.134	21	39
Si-AGO2	Si000241m	902	101	9.36	78.647	0.460	0.279	0.104	0.133	25	35
Si-AGO3	Si034423m	730	82.79	9.31	79.658	0.463	0.278	0.104	0.134	17	36
Si-AGO4	Si040253m	1030	115.461	9.62	82.155	0.461	0.280	0.101	0.132	37	32
Si-AGO5	Si034005m	1094	118	9.75	77.176	0.460	0.282	0.102	0.130	28	42
Si-AGO6	Si016190m	1023	113.72	9.62	77.722	0.467	0.269	0.109	0.136	31	29
Si-AGO7	Si009227m	1083	120	9.56	72	0.444	0.289	0.111	0.129	25	38
Si-AGO8	Si009244m	1024	110.78	9.4	75.6	0.485	0.267	0.093	0.135	25	29
Si-AGO9	Si021147m.1	910	101.6	9.22	79.13	0.460	0.277	0.102	0.135	22	37
Si-AGO10	Si021039m.1	1102	121	9.65	73.66	0.504	0.269	0.083	0.118	27	34
Si-AGO11	Si005792m	959	106.97	9.53	82.471	0.500	0.273	0.083	0.118	27	39
Si-AGO12	Si005737m	1098	122.35	9.41	73.379	0.465	0.299	0.092	0.116	34	32
Si-AGO13	Si028876m	888	98.604	8.99	83.547	0.472	0.290	0.089	0.118	30	36
Si-AGO14	Si028918m	846	94.66	9.53	86.93	0.463	0.298	0.094	0.118	20	38
Si-AGO15	Si028786m	1041	112.64	9.59	73.67	0.466	0.303	0.089	0.115	20	40
Me-AGO1	cassava4.1_028612m	922	104	9.38	83.94	0.463	0.282	0.099	0.132	27	41

Me-AGO2	cassava4.1_000932m	993	111	9.4	78.2	0.465	0.275	0.100	0.131	36	34
Me-AGO3	cassava4.1_000946m	990	111	9.47	78.495	0.466	0.270	0.100	0.135	35	30
Me-AGO4	cassava4.1_000940m	991	110	9.68	75.53	0.460	0.281	0.097	0.137	32	35
Me-AGO5	cassava4.1_000956m	988	110	9.49	77.9	0.462	0.280	0.10	0.133	28	40
Me-AGO6	cassava4.1_028537m	942	104.5	9.29	69.13	0.482	0.274	0.094	0.121	20	32
Me-AGO7	cassava4.1_000845m	1017	115	9.59	80.51	0.456	0.280	0.095	0.135	34	33
Me-AGO8	cassava4.1_000826m	1021	116	9.5	82.292	0.452	0.294	0.095	0.130	31	36
Me-AGO9	cassava4.1_001305m	909	101	9.25	80.92	0.468	0.268	0.110	0.131	19	40
Me-AGO10	cassava4.1_001312m	908	102	9.24	83.051	0.474	0.264	0.110	0.130	22	43
Me-AGO11	cassava4.1_000920m	995	110	9.51	77.095	0.485	0.254	0.099	0.131	26	40
Me-AGO12	cassava4.1_001630m	845	95	9.06	85.219	0.466	0.266	0.114	0.130	22	43
Me-AGO13	cassava4.1_021803m	683	76	9.75	83.47	0.471	0.264	0.102	0.148	16	28
At-AGO1	AT1G48410.2	1050	116.459	9.5	74.724	0.477	0.278	0.093	0.125	30	33
At-AGO2	AT1G31280.1	1014	113.422	9.65	70.227	0.477	0.242	0.113	0.148	22	45
At-AGO3	AT1G31290.1	1194	129.18	9.51	70.46	0.49	0.243	0.110	0.135	23	51
At-AGO4	AT2G27040.1	924	102.83	9.25	80.27	0.476	0.275	0.104	0.123	26	42
At-AGO5	AT2G27880.1	997	111.087	9.63	78.857	0.454	0.287	0.10	0.136	26	38
At-AGO6	AT2G32940.1	878	98.68	8.92	84.681	0.475	0.264	0.110	0.124	15	47
At-AGO7	AT1G69440.1	990	113.395	9.43	81.98	0.449	0.278	0.100	0.133	33	31
At-AGO8	AT5G21030.1	850	95.506	9.18	82.435	0.462	0.293	0.096	0.118	20	36
At-AGO9	AT5G21150.1	896	100.523	9.35	80.692	0.479	0.277	0.099	0.124	23	38
At-AGO10	AT5G43810.1	988	110.866	9.51	80.192	0.465	0.277	0.098	0.135	31	38
Cc-AGO1	Ciclev10003971m	723	81.436	9.91	78.465	0.472	0.272	0.093	0.138	18	28
Cc-AGO2	Ciclev10004245m	992	111.541	9.49	79.889	0.460	0.281	0.098	0.133	36	31
Cc-AGO3	Ciclev10013619m	453	51.129	9.91	84.79	0.494	0.256	0.084	0.139	12	22
Cc-AGO4	Ciclev10011166m	730	82.062	9.45	81.479	0.473	0.255	0.107	0.141	25	27
Cc-AGO5	Ciclev10030593m	1030	117.063	9.37	81.01	0.450	0.293	0.094	0.125	33	31
Cc-AGO6	Ciclev10018625m.1	1073	118.306	9.47	72.022	0.483	0.271	0.093	0.122	24	38

Cc-AGO7	Ciclev10027752m	973	108.054	9.1	81.151	0.479	0.282	0.099	0.114	20	49
Cc-AGO8	Ciclev10027750m	975	108.576	9.74	78.892	0.479	0.281	0.088	0.125	25	45
Cc-AGO9	Ciclev10027755m	963	106.981	9.59	80.363	0.476	0.286	0.090	0.123	26	51
Cc-AGO10	Ciclev10027763m	955	106.928	9.36	82.283	0.476	0.286	0.095	0.119	26	41
Cc-AGO11	Ciclev10027760m	960	106.689	9.51	82.031	0.480	0.283	0.091	0.120	21	56
Cc-AGO12	Ciclev10014153m	981	110	9.29	80	0.476	0.286	0.090	0.123	23	40
Cc-AGO13	Ciclev10014144m	991	111.051	9.43	75.974	0.487	0.245	0.106	0.134	21	50
Cc-AGO14	Ciclev10014201m.1	898	100.919	9.54	86.281	0.472	0.273	0.100	0.131	21	34
Cc-AGO15	Ciclev10014186m	920	103.012	9.17	83.12	0.472	0.268	0.111	0.128	20	38
Pvi-AGO1	Pavirv00034080m	1,032	112.486	9.33	66.986	0.497	0.252	0.100	0.127	19	48
Pvi-AGO2	Pavirv00030355m	1,108	122.074	9.58	72.491	0.489	0.276	0.088	0.121	27	32
Pvi-AGO3	Pavirv00009860m	1,006	109.981	9.7	76.799	0.510	0.258	0.081	0.120	28	39
Pvi-AGO4	Pavirv00008497m	899	102.301	9.33	83.904	0.453	0.267	0.106	0.133	30	31
Pvi-AGO5	Pavirv00009309m	1,048	114.334	9.45	68.464	0.497	0.243	0.100	0.133	26	47
Pvi-AGO6	Pavirv00009308m	931	104.035	9.2	81.708	0.472	0.252	0.112	0.134	33	36
Pvi-AGO7	Pavirv00010118m.1	1,070	118.758	9.59	74.243	0.485	0.267	0.093	0.125	25	37
Pvi-AGO8	Pavirv00026903m.1	821	92.994	9.23	86.687	0.475	0.267	0.102	0.128	22	34
Pvi-AGO9	Pavirv00070119m	854	95.823	9.34	87.494	0.467	0.272	0.104	0.133	24	34
Pvi-AGO10	Pavirv00062436m	906	101	9.11	79.581	0.471	0.259	0.113	0.129	29	36
Pvi-AGO11	Pavirv00024831m	866	97.58	9.45	86.52	0.462	0.281	0.103	0.132	31	32
Pvi-AGO12	Pavirv00047388m	902	100	9.3	80.71	0.466	0.271	0.10	0.132	27	35
Pvi-AGO13	Pavirv00044789m	635	71.735	9.41	81.055	0.465	0.272	0.099	0.134	21	23
Pvi-AGO14	Pavirv00060575m	912	102.189	9.21	82.895	0.482	0.237	0.115	0.136	28	32
Pvi-AGO15	Pavirv00064834m	1027	114.27	9.58	76.553	0.485	0.257	0.096	0.129	29	31
Pvi-AGO16	Pavirv00069294m	1049	116.6	9.37	75.968	0.484	0.257	0.100	0.126	30	32
Pvi-AGO17	Pavirv00005128m	1035	116	9.6	83.64	0.487	0.245	0.096	0.133	32	35
Pvi-AGO18	Pavirv00026790m	868	94.30	9.96	77.6	0.516	0.251	0.083	0.132	25	25
Pvi-AGO19	Pavirv00037459m	871	97.468	9.23	80.23	0.472	0.258	0.113	0.133	26	31
Pvi-AGO20	Pavirv00020679m	773	86.612	9.16	86.973	0.471	0.269	0.106	0.128	23	30

Pvi-AGO21	Pavirv00037206m	1034	114.93	9.24	75.658	0.482	0.262	0.102	0.123	32	31
Pvi-AGO22	Pavirv00057838m	899	100	9.31	79.68	0.471	0.260	0.111	0.135	30	37
Pvi-AGO23	Pavirv00063222m	802	90.924	9.34	87.606	0.465	0.268	0.106	0.130	27	35
Pvi-AGO24	Pavirv00033214m	805	91.22	9.31	86.112	0.473	0.270	0.103	0.127	24	33
Glym-AGO1	Glyma05g08170.2	908	102.846	9.28	88.337	0.463	0.270	0.106	0.134	31	38
Glym-AGO2	Glyma17g12850.2	904	102.534	9.21	87.544	0.459	0.275	0.107	0.134	32	38
Glym-AGO3	Glyma06g23920.3	909	103	9.3	86	0.459	0.275	0.105	0.134	29	45
Glym-AGO4	Glyma04g21450.2	909	103.21	9.31	87.16	0.46	0.278	0.103	0.133	27	43
Glym-AGO5	Glyma20g28970.1	974	109.514	9.4	79.07	0.462	0.279	0.102	0.136	36	38
Glym-AGO6	Glyma02g00510.1	972	109	9.39	81.821	0.473	0.269	0.099	0.133	35	38
Glym-AGO7	Glyma10g38770.1	974	109	9.38	78.9	0.462	0.277	0.103	0.134	36	32
Glym-AGO8	Glyma10g00527.1	949	106.678	9.43	81.834	0.467	0.276	0.099	0.134	31	38
Glym-AGO9	Glyma16g34300.1	1053	116.503	9.47	73.78	0.49	0.264	0.094	0.124	28	38
Glym-AGO10	Glyma09g29720.1	1058	116	9.48	73	0.495	0.261	0.094	0.125	28	35
Glym-AGO11	Glyma12g08860.1	961	107.559	9.74	80.42	0.47	0.27	0.095	0.134	31	39
Glym-AGO12	Glyma11g19650.2	890	99.58	9.79	81.921	0.473	0.258	0.097	0.138	24	32
Glym-AGO13	Glyma02g12430.2	1031	117.896	9.38	83.27	0.440	0.288	0.098	0.129	30	35
Glym-AGO14	Glyma01g06370.2	1029	117.63	9.37	83.061	0.443	0.289	0.096	0.125	32	37
Glym-AGO15	Glyma20g12070.3	947	106	9.15	81.595	0.476	0.259	0.112	0.129	19	35
Glym-AGO16	Glyma02g44260.1	906	101.152	9.23	83.466	0.478	0.265	0.109	0.129	26	37
Glym-AGO17	Glyma14g04510.1	906	101.416	9.26	82.274	0.475	0.265	0.110	0.131	23	32
Glym-AGO18	Glyma13g26240.2	913	101	9.02	92.421	0.470	0.279	0.103	0.118	21	40
Glym-AGO19	Glyma06g47230.1	881	99.7	9.51	84.60	0.461	0.278	0.101	0.135	21	37
Glym-AGO20	Glyma15g13260.2	1037	117.38	9.1	80.347	0.474	0.261	0.112	0.131	26	39
Glym-AGO21	Glyma20g02820.1	966	108.51	9.47	77.919	0.461	0.285	0.097	0.131	19	46
Br-AGO1	Bra040815	1009	112.539	9.66	73.013	0.475	0.245	0.114	0.146	34	40
Br-AGO2	Bra032254	1111	122.885	9.53	70.027	0.481	0.276	0.092	0.125	31	33
Br-AGO3	Bra020152	906	101.33	9.43	81.832	0.470	0.288	0.097	0.123	27	33

Br-AGO4	Bra002360	930	105.373	9.37	81.828	0.471	0.276	0.100	0.126	21	42
Br-AGO5	Bra002349	1701	190.59	8.95	85.209	0.481	0.256	0.115	0.126	49	74
Br-AGO6	Bra002361	911	102.278	9.44	79.978	0.473	0.276	0.101	0.128	22	44
Br-AGO7	Bra033698	974	109.128	9.51	80.575	0.476	0.264	0.099	0.134	34	35
Br-AGO8	Bra003999	981	112.137	9.5	82.11	0.446	0.278	0.100	0.136	33	39
Br-AGO9	Bra011993	966	107.703	9.64	79.524	0.468	0.272	0.101	0.139	32	32
Br-AGO10	Bra022918	867	97.216	9.21	86.424	0.475	0.271	0.106	0.127	19	38
Br-AGO11	Bra023172	1037	115.561	9.79	71.398	0.471	0.252	0.110	0.148	25	50
Br-AGO12	Bra034318	922	103.032	9.06	79.599	0.476	0.273	0.106	0.121	24	40
Br-AGO13	Bra014136	1079	119.424	9.52	71.103	0.482	0.270	0.094	0.126	28	32
Vv-AGO1	GSVIVT01001941001	881	98.98	9.6	79.444	0.467	0.293	0.085	0.123	20	39
Vv-AGO2	GSVIVT01012490001	889	101.71	9.63	81.249	0.457	0.265	0.107	0.142	35	20
Vv-AGO3	GSVIVT01012529001	695	78.86	9.67	80.662	0.459	0.282	0.096	0.135	23	18
Vv-AGO4	GSVIVT01014252001	442	49.706	8.98	89.095	0.493	0.269	0.097	0.113	14	18
Vv-AGO5	GSVIVT01015464001	905	101.78	9.28	86.133	0.463	0.280	0.103	0.130	24	43
Vv-AGO6	GSVIVT01018054001	953	107	9.38	79.37	0.465	0.273	0.102	0.132	35	34
Vv-AGO7	GSVIVT01025868001	879	98.15	9.24	81.1	0.466	0.283	0.102	0.123	20	39
Vv-AGO8	GSVIVT01026261001	983	109	9.6	81.974	0.503	0.250	0.094	0.131	20	49
Vv-AGO9	GSVIVT01026264001	978	109.03	9.31	80.951	0.491	0.258	0.097	0.128	17	49
Vv-AGO10	GSVIVT01026268001	994	112	9.59	81.469	0.490	0.234	0.106	0.142	28	43
Vv-AGO11	GSVIVT01029383001	1038	115.40	9.43	76.657	0.480	0.272	0.096	0.125	30	32
Vv-AGO12	GSVIVT01030512001	900	100	9.41	87.156	0.473	0.267	0.102	0.131	18	45
Vv-AGO13	GSVIVT01031430001	1032	114.458	9.72	75.155	0.468	0.280	0.094	0.134	26	44
Vv-AGO14	GSVIVT01033726001	851	97.02	9.02	88.061	0.488	0.256	0.106	0.123	23	40
Vv-AGO15	GSVIVT01037488001	913	101	9.26	82.486	0.473	0.268	0.108	0.128	20	39
Ppr-AGO1	ppa000990m	939	104.195	9.06	82.694	0.482	0.265	0.109	0.122	18	45
Ppr-AGO2	ppa024131m	1,003	111.925	9.58	76.401	0.475	0.263	0.101	0.133	35	36
Ppr-AGO3	ppa000759m	1,013	114.939	9.43	82.359	0.457	0.283	0.100	0.131	31	35

Ppr-AGO4	ppa025242m	872	97.831	9.56	83.016	0.474	0.274	0.099	0.131	20	37
Ppr-AGO5	ppa000547m	1,102	121.592	9.5	70.998	0.487	0.274	0.091	0.121	29	32
Ppr-AGO6	ppa026254m	938	104.702	9.09	83.731	0.480	0.275	0.103	0.123	25	43
Ppr-AGO7	ppa000619m.1	1,069	118.535	9.46	72.498	0.484	0.266	0.097	0.127	26	36
Ppr-AGO8	ppa017623m	911	101.678	9.43	84.61	0.484	0.268	0.100	0.127	22	40
Ppr-AGO9	ppa001137m	898	100.254	9.16	85.824	0.474	0.278	0.101	0.121	19	37
Ppr-AGO10	ppa000823m	990	111.243	9.43	79.273	0.470	0.272	0.098	0.132	33	33
Ppr-AGO11	ppa000866m	976	108.594	9.47	78.678	0.481	0.257	0.100	0.132	24	31
Bd-AGO1	Bradi2g10360.1	638	70.892	8.97	84.436	0.502	0.255	0.096	0.110	11	34
Bd-AGO2	Bradi2g14147.1	914	101.853	9.36	84.989	0.486	0.246	0.109	0.133	25	46
Bd-AGO3	Bradi2g10370.1	924	102.862	9.15	78.983	0.473	0.267	0.108	0.127	23	39
Bd-AGO4	Bradi4g08587.1	882	98.259	9.39	84.841	0.477	0.262	0.104	0.132	19	39
Bd-AGO5	Bradi1g12430.1	766	85.497	9.2	91.253	0.491	0.269	0.099	0.123	27	27
Bd-AGO6	Bradi1g05162.1	1070	117.79	9.38	76.645	0.514	0.244	0.095	0.122	27	50
Bd-AGO7	Bradi1g28260.1	1075	118.398	9.4	70.242	0.475	0.273	0.098	0.130	21	47
Bd-AGO8	Bradi1g16060.1	1038	116.578	9.56	80.472	0.474	0.262	0.096	0.134	32	36
Bd-AGO9	Bradi1g36907.1	953	106.573	9.5	82.371	0.490	0.250	0.099	0.134	26	44
Bd-AGO10	Bradi1g54977.1	1029	111.4	9.64	76.181	0.520	0.250	0.085	0.122	29	39
Bd-AGO11	Bradi1g29577.1	1044	116.194	9.22	79.023	0.483	0.261	0.102	0.122	34	24
Bd-AGO12	Bradi5g18540.1	1094	121.346	9.57	72.888	0.486	0.266	0.093	0.126	27	39
Bd-AGO13	Bradi5g21810.1	899	101.484	9.4	74.905	0.446	0.254	0.120	0.150	34	21
Bd-AGO14	Bradi5g21800.1	1027	111.399	9.44	67.118	0.500	0.250	0.098	0.129	28	26
Bd-AGO15	Bradi3g51077.1	1053	117.441	9.72	84.881	0.484	0.270	0.088	0.125	27	47
Bd-AGO16	Bradi3g60697.1	1043	115.98	9.22	79.023	0.483	0.261	0.102	0.122	31	33
Cs-AGO1	Cucsa.082260.1	926	105.126	9.34	84.633	0.459	0.280	0.102	0.133	32	38
Cs-AGO2	Cucsa.112480.1	1058	117.418	9.41	71.862	0.470	0.278	0.095	0.123	27	36
Cs-AGO3	Cucsa.152920.1	915	102.07	9.25	83.596	0.480	0.268	0.106	0.127	20	41
Cs-AGO4	Cucsa.185140.1	904	101.508	9.36	85.277	0.470	0.271	0.104	0.132	18	44

Cs-AGO5	Cucsa.200260.1	860	96.869	9.27	83.14	0.472	0.269	0.102	0.128	20	49
Cs-AGO6	Cucsa.254700.1	984	110.71	9.51	79.451	0.467	0.271	0.101	0.137	34	32
Cs-AGO7	Cucsa.284770.1	1019	115.9	9.39	84.328	0.467	0.272	0.097	0.129	26	43
Th-AGO1	Thhalv10016181m	989	109.369	9.68	77.169	0.466	0.281	0.096	0.133	27	34
Th-AGO2	Thhalv10016224m	877	99	9.3	83.888	0.491	0.264	0.098	0.123	22	40
Th-AGO3	Thhalv10011196m.1	1084	119.4	9.54	72.214	0.492	0.264	0.096	0.123	28	36
Th-AGO4	Thhalv10006643m	1058	117	9.75	67.401	0.493	0.264	0.097	0.121	23	51
Th-AGO5	Thhalv10018068m	999	114.24	9.49	80.751	0.447	0.286	0.100	0.133	29	37
Th-AGO6	Thhalv10001902m	922	103.19	9.03	79.924	0.478	0.270	0.101	0.127	22	37
Th-AGO7	Thhalv10015501m	850	96.037	9.49	82.071	0.470	0.275	0.103	0.128	26	46
Th-AGO7	Thhalv10012624m	903	100.63	9.5	83.27	0.459	0.288	0.103	0.123	25	40
Th-AGO9	Thhalv10003139m	979	109	9.55	80.746	0.460	0.278	0.103	0.133	33	34
St-AGO1	PGSC0003DMT400053301.1	920	104	9.36	84.13	0.467	0.298	0.090	0.117	33	39
St-AGO2	PGSC0003DMT400069313.1	1054	117	9.53	74.35	0.475	0.290	0.092	0.119	32	32
St-AGO3	PGSC0003DMT400018363.1	1025	114	9.45	74.63	0.473	0.288	0.091	0.126	24	50
St-AGO4	PGSC0003DMT400069459.1	913	101.9	9.15	81.7	0.475	0.291	0.091	0.122	19	41
St-AGO5	PGSC0003DMT400077569	959	107	9.44	79.99	0.465	0.289	0.102	0.115	25	35
St-AGO6	PGSC0003DMT400079716.1	1127	124	9.56	70.5	0.472	0.300	0.087	0.120	33	31
St-AGO7	PGSC0003DMT400062439	1002	114.23	9.36	85.96	0.468	0.284	0.102	0.118	30	34
St-AGO8	PGSC0003DMT400058471.1	909	101.7	9.21	81.45	0.467	0.286	0.102	0.116	18	45
St-AGO9	PGSC0003DMT400054669	1051	117	9.47	72.1	0.451	0.298	0.104	0.115	23	35
St-AGO10	PGSC0003DMT400054667	730	82.9	8.77	85.65	0.492	0.277	0.085	0.115	23	28
St-AGO11	PGSC0003DMT400054661	980	110	9.36	77.347	0.489	0.278	0.087	0.117	24	37
Cp-AGO1	supercontig_1.68	879	99.937	9.37	84.835	0.469	0.276	0.101	0.129	28	29
Cp-AGO2	supercontig_135.40	1012	114.03	9.25	76.808	0.458	0.263	0.110	0.135	20	48
Cp-AGO3	supercontig_26.59	923	103.149	9.13	84.269	0.481	0.258	0.112	0.128	20	42
Cp-AGO4	supercontig_44.130	990	111.617	9.38	78.384	0.462	0.274	0.103	0.134	34	32

Cp-AGO5	supercontig_47.31	886	100.145	9.37	85.734	0.468	0.278	0.102	0.130	29	35
Cp-AGO6	supercontig_75.90	915	103.738	9.25	83.625	0.445	0.275	0.102	0.128	26	43
Os-AGO1	LOC_Os04g52550.1	1109	122	9.37	72.29	0.463	0.241	0.118	0.147	29	37
Os-AGO2	LOC_Os04g47870.1	1118	123.59	9.6	72.8	0.487	0.267	0.089	0.122	24	38
Os-AGO3	LOC_Os04g06770.2.1	911	101	9.26	81.3	0.473	0.267	0.108	0.128	29	35
Os-AGO4	LOC_Os07g09020.1	1052	113	9.72	76.673	0.519	0.258	0.081	0.120	25	47
Os-AGO5	LOC_Os01g16870.3.1	904	100	9.31	81.62	0.475	0.265	0.107	0.131	27	35
Os-AGO6	LOC_Os03g47830.1	892	98.7	8.75	77.8	0.478	0.262	0.111	0.121	24	28
Os-AGO7	LOC_Os03g57560.1	1060	121	9.44	86.33	0.468	0.262	0.101	0.137	36	46
Os-AGO8	LOC_Os03g47820.1	1058	116	9.59	75	0.483	0.270	0.092	0.131	27	43
Os-AGO9	LOC_Os03g58600.1	1058	117	9.42	75.46	0.499	0.250	0.098	0.127	24	50
Os-AGO10	LOC_Os03g33650.1	1048	117	9.58	84.17	0.487	0.240	0.101	0.137	35	35
Os-AGO11	LOC_Os02g58490.1	1011	113	9.65	79.01	0.476	0.271	0.093	0.130	30	35
Os-AGO12	LOC_Os02g45070.1	1082	120	9.52	74.9	0.482	0.27	0.092	0.122	31	39
Os-AGO13	LOC_Os02g07310.1	876	98	9.09	88	0.473	0.274	0.098	0.120	33	36
Os-AGO14	LOC_Os06g51310.2.1	1038	115	9.28	77.52	0.479	0.266	0.098	0.127	37	24
Os-AGO15	LOC_Os06g39640.1	973	107	9.5	79.99	0.491	0.254	0.096	0.131	24	42
AC-AGO1	Aquca_014_00920.1	835	93.659	9.42	83.593	0.471	0.275	0.103	0.128	26	32
AC-AGO2	Aquca_014_00922.1	905	100.793	9.45	83.348	0.492	0.264	0.098	0.123	25	35
AC-AGO3	Aquca_035_00087.1	898	100.708	9.55	86.715	0.479	0.263	0.102	0.134	29	32
AC -AGO4	Aquca_002_01245.1	1049	118.209	9.32	91.23	0.485	0.268	0.093	0.135	24	56
AC -AGO5	Aquca_006_00113.1	785	88.582	9.66	90.484	0.485	0.268	0.093	0.135	21	38
AC -AGO6	Aquca_013_00448.1	876	99.446	9.31	83.436	0.474	0.255	0.107	0.132	17	46
AC -AGO7	Aquca_002_00494.1	663	74.211	9.78	87.315	0.469	0.270	0.100	0.149	20	25
AC -AGO8	Aquca_025_00079.1	898	100.91	9.28	89.833	0.474	0.265	0.108	0.131	23	34
AC -AGO9	Aquca_091_00081.1	1084	120.186	9.56	71.762	0.473	0.282	0.091	0.125	29	33
AC -AGO10	Aquca_022_00300.1	920	103.04	9.61	81.37	0.460	0.277	0.097	0.134	35	38
AC -AGO11	Aquca_017_00554.1	999	112.323	9.33	80.09	0.460	0.278	0.103	0.133	34	41

AC -AGO12	Aquca_001_00697.1	941	107.081	9.38	84.208	0.460	0.277	0.102	0.135	36	32
AC -AGO13	Aquca_002_01350.1	1054	115.479	9.67	76.66	0.505	0.269	0.083	0.118	28	35
AC -AGO14	Aquca_002_00707.1	952	107.86	9.44	85.903	0.458	0.287	0.100	0.129	34	27
AC -AGO15	Aquca_002_01349.1	1038	115.599	9.28	74.335	0.465	0.300	0.092	0.117	20	41
AC -AGO16	Aquca_017_00554.1	999	112.323	9.33	80.09	0.460	0.278	0.103	0.133	34	41
AC -AGO17	Aquca_002_01350.1	1054	115.479	9.67	76.66	0.505	0.269	0.083	0.118	28	35
AC -AGO18	Aquca_019_00127.1	917	102.708	9.58	81.614	0.463	0.281	0.095	0.130	27	37
Pt-AGO1	Potri.010G081300.1	999	111.937	9.48	79.429	0.465	0.276	0.097	0.133	33	36
Pt-AGO2	Potri.010G163800.1	1,030	117.405	9.51	81.573	0.455	0.290	0.093	0.130	36	34
Pt-AGO3	Potri.009G001500.1	987	110.386	9.56	77.649	0.466	0.273	0.101	0.139	26	38
Pt-AGO4	Potri.006G118600.1	879	98.054	9.47	83.641	0.481	0.272	0.093	0.127	28	34
Pt-AGO5	Potri.006G025900.1	930	103.745	9.1	82.269	0.477	0.278	0.103	0.118	22	36
Pt-AGO6	Potri.016G024200.1	921	102.817	9.2	81.292	0.479	0.277	0.102	0.119	25	31
Pt-AGO7	Potri.015G029000.1	875	98.738	9.41	83.669	0.480	0.256	0.102	0.133	25	35
Pt-AGO8	Potri.015G117400.1	895	101.168	9.35	85.777	0.468	0.268	0.104	0.132	23	42
Pt-AGO9	Potri.014G159400.1	910	101.656	9.32	84.473	0.470	0.279	0.100	0.124	24	38
Pt-AGO10	Potri.008G010500.1	923	103.479	9.19	80.856	0.463	0.282	0.104	0.124	18	43
Pt-AGO11	Potri.008G158800.1	996	111.731	9.38	79.488	0.463	0.277	0.100	0.132	34	32
Pt-AGO12	Potri.001G213700.1	985	110.131	9.59	75.411	0.456	0.277	0.102	0.138	29	37
Pt-AGO13	Potri.001G219700.1	911	101.825	9.18	82.119	0.480	0.265	0.108	0.126	23	37
Pt-AGO14	Potri.012G118700.1	1,039	113.807	9.4	72.936	0.494	0.235	0.108	0.137	19	46
Pt-AGO15	Potri.012G037100.1	1,062	117.665	9.48	73.531	0.484	0.266	0.097	0.128	27	36
Md-AGO1	MDP0000285251	2583	288.42	9.19	82	0.474	0.292	0.091	0.113	71	99
Md-AGO2	MDP0000161046	1115	121	9.37	69.27	0.493	0.277	0.091	0.116	28	35
Md-AGO3	MDP0000118779	1016	114	9.48	84.204	0.461	0.270	0.100	0.132	35	30
Md-AGO4	MDP0000215105	845	94.75	9.53	79.709	0.463	0.270	0.102	0.130	18	39
Md-AGO5	MDP0000292511	2251	253	9.03	81.51	0.472	0.260	0.115	0.131	61	84
Md-AGO6	MDP0000071268	988	110	9.45	80.73	0.474	0.268	0.096	0.131	34	32

Md-AGO7	MDP0000159246	1024	116	9.49	84.355	0.464	0.277	0.097	0.130	40	31
Md-AGO8	MDP0000069525	1093	121	9.43	72.608	0.483	0.274	0.092	0.123	29	37
Md-AGO9	MDP0000209079	888	99	9.51	83.4	0.475	0.264	0.104	0.134	25	32
Md-AGO10	MDP0000774227	982	109	9.65	76.65	0.476	0.259	0.101	0.138	36	35
Md-AGO11	MDP0000232035	978	109	9.7	78.646	0.480	0.258	0.096	0.136	34	31
Md-AGO12	MDP0000199819	974	109.	9.44	83.19	0.477	0.265	0.095	0.129	33	34
Md-AGO13	MDP0000305971	2043	227.855	8.84	83.19	0.483	0.276	0.098	0.111	61	79
Md-AGO14	MDP0000191579	956	106	9.35	81.419	0.476	0.270	0.097	0.128	32	33
Md-AGO15	MDP0000260407	994	109	9.31	75.0	0.501	0.262	0.089	0.115	24	37
Gr-AGO1	Gorai.010G171800.1	1028	115.56	9.36	76.411	0.484	0.262	0.101	0.129	20	47
Gr-AGO2	Gorai.009G325500.1	1015	115	9.46	82	0.466	0.275	0.096	0.129	28	33
Gr-AGO3	Gorai.009G446700.1	1034	115.014	9.42	75.319	0.468	0.273	0.102	0.132	22	39
Gr-AGO4	Gorai.009G021900.1	937	105	9.29	83.93	0.460	0.279	0.105	0.133	28	48
Gr-AGO5	Gorai.009G105600.1	912	102	9.32	82.05	0.468	0.285	0.102	0.124	20	42
Gr-AGO6	Gorai.004G228100.1	913	101	9.18	81.961	0.469	0.283	0.105	0.124	22	35
Gr-AGO7	Gorai.013G089400.1	1006	114	9.47	79.1	0.454	0.288	0.094	0.128	30	35
Gr-AGO8	Gorai.011G166600.1	995	111	9.45	78.09	0.462	0.280	0.097	0.131	34	32
Gr-AGO9	Gorai.008G294100.1	1080	119	9.48	73.565	0.489	0.268	0.094	0.124	26	37
Gr-AGO10	Gorai.001G195700.1	898	100	9.36	89.54	0.472	0.267	0.107	0.135	28	34
Gr-AGO11	Gorai.006G189400.1	1053	116	9.4	74.2	0.481	0.273	0.093	0.124	28	36
Gr-AGO12	Gorai.006G103100.1	939	104	9.45	79.27	0.486	0.275	0.092	0.129	24	41
Gr-AGO13	Gorai.006G243200.1	953	106	9.43	79.087	0.465	0.281	0.097	0.130	32	32
